# Adaptation of Phenylalanine and Tyrosine Catabolic Pathway to Hibernation in Bats

**DOI:** 10.1371/journal.pone.0062039

**Published:** 2013-04-19

**Authors:** Yi-Hsuan Pan, Yijian Zhang, Jie Cui, Yang Liu, Bronwyn M. McAllan, Chen-Chung Liao, Shuyi Zhang

**Affiliations:** 1 Laboratory of Molecular Ecology and Evolution, Institute for Advanced Studies in Multidisciplinary Science and Technology, East China Normal University, Shanghai, China; 2 Discipline of Physiology and Bosch Institute, School of Medical Science, The University of Sydney, Sydney, New South Wales, Australia; 3 Proteomic Research Center, National Yang-Ming University, Taipei, Taiwan; University of Sydney, Australia

## Abstract

Some mammals hibernate in response to harsh environments. Although hibernating mammals may metabolize proteins, the nitrogen metabolic pathways commonly activated during hibernation are not fully characterized. In contrast to the hypothesis of amino acid preservation, we found evidence of amino acid metabolism as three of five key enzymes, including phenylalanine hydroxylase (PAH), homogentisate 1,2-dioxygenase (HGD), fumarylacetoacetase (FAH), involved in phenylalanine and tyrosine catabolism were co-upregulated during hibernation in two distantly related species of bats, *Myotis ricketti* and *Rhinolophus ferrumequinum*. In addition, the levels of phenylalanine in the livers of these bats were significantly decreased during hibernation. Because phenylalanine and tyrosine are both glucogenic and ketogenic, these results indicate the role of this catabolic pathway in energy supply. Since any deficiency in the catabolism of these two amino acids can cause accumulations of toxic metabolites, these results also suggest the detoxification role of these enzymes during hibernation. A higher selective constraint on *PAH*, *HPD*, and *HGD* in hibernators than in non-hibernators was observed, and hibernators had more conserved amino acid residues in each of these enzymes than non-hibernators. These conserved amino acid residues are mostly located in positions critical for the structure and activity of the enzymes. Taken together, results of this work provide novel insights in nitrogen metabolism and removal of harmful metabolites during bat hibernation.

## Introduction

Hibernation is a controlled reversible reduction of body temperature, metabolic rate, and many other physiological processes of some animals in order to survive in winter [1]–[4]. Understanding the mechanisms by which hibernators control their metabolism during hibernation to cope with stress conditions, such as hypothermia, ischemia, and muscle atrophy, may aid in the prevention or treatment of human diseases. A winter hibernation consists of a sequence of torpor bouts lasting for days or weeks interrupted by short normothermic arousal bouts [1], [2]. In preparation for hibernation, the weight and fat mass of some hibernators (e.g., squirrels, bats, and black bears) are greatly increased in summer and autumn [1]. During hibernation, many hibernators do not feed and depend largely on fat reserves for energy by switching from glucose utilization to lipid consumption [1], [3].

In large hibernators (e.g., bears), only a small loss of proteins occurs during hibernation [5], [6]. Although small hibernators (e.g., bats and squirrels) lose a significant overall protein mass during hibernation [7]–[10], little loss of pectoral and biceps brachii muscles or liver proteins is observed [11], [12]. Several mechanisms that may prevent muscle atrophy or loss of liver proteins have been proposed, including the production of proteolytic inhibitors [5], [6], continual protein synthesis [12], [13], maintenance of proper protein folding [11], and preservation of essential amino acids such as phenylalanine (Phe) and tyrosine (Tyr) [12]–[14]. These two amino acids are both glucogenic and ketogenic in energy production. They are also precursors of thyroid hormones and catecholamines (e.g., adrenaline, noradrenaline, and dopamine) that can act on adipose tissues to modulate thermal and energy balance during hibernation [15], [16].

In mammals, tyrosine is synthesized from phenylalanine. Conversion of phenylalanine to tyrosine is an irreversible step catalyzed by phenylalanine hydroxylase (PAH) [17], [18]. Four enzymes including tyrosine aminotransferase (TAT), hydroxyphenylpyruvate dioxygenase (HPD) [19], homogentisate 1,2-dioxygenase (HGD) [20], [21], and fumarylacetoacetase (FAH) are involved in tyrosine catabolism [22]–[24]. Any deficiency in these enzymes may result in accumulations of toxic metabolites such as 4-hydroxyphenylpyruvate, homogentisate, 4-maleylacetoacetate, and fumarylacetate in blood, liver, or urine leading to DNA and tissue damage, depletion of glutathione levels, and activation of apoptosis [25], [26].

Although hibernators undergo tremendous alterations in physiology with many genes differentially expressed during hibernation [1], there has been no evolutionary evidence for the adaptation of an entire metabolic pathway to hibernation. Bats represent approximately twenty percent of all mammalian species and are the only flying mammals that hibernate. Their physiological status during hibernation has been extensively studied [27]–[30]. Taxonomically, bats belong to the order Chiroptera that is subdivided into suborders Yinpterochiroptera and Yangochiroptera. Hibernating bats are found in both suborders (e.g., *Rhinolophus* in Yinpterochiroptera and *Myotis* in Yangochiroptera suborder), while most non-hibernating bats are in the Yinpterochiroptera suborder (e.g., *Cynopterus*) [27], [31].

Bats offer the unique advantage over bears and squirrels for investigation of enzymes in adaptation to hibernation as there are at least 1,200 species of bats, and the phylogenetic relationships of most of these bats are well defined [31]. A careful comparison of the activities, expression levels, and structures of enzymes between different hibernating and non-hibernating species of bats will yield useful information in the regulation and evolution of the enzymes. The adaptation of liver proteins in 13-lined ground squirrels to hibernation has been investigated using proteomic approaches [12]. In this study, we used a combination of proteomic and evolutional approaches to investigate the adaptation of PAH, HPD, HGD, and FAH in the phenylalanine and tyrosine catabolic pathway to hibernation.

## Materials and Methods

### Animal and Tissue Acquisition

Acquisition of bats and all experiments involved animals were done in accordance with the Guidelines and Regulations for the Use of Laboratory Animals (Decree No. 2, State Science and Technology Commission, People’s Republic of China, November 14, 1988). Animal use protocols for this study were approved by the Animal Ethics Committee, East China Normal University (ID no: 20090219 and 20101002).

Non-hibernating *Cynopterus sphinx* and *Rousettus leschenaultii* bats were captured from Maoming, Guangdong Province (21°39′N, 110°55′E) and Mashan County, Guangxi (23°55′N, 108°26′E) Province, China, respectively. Hibernating *Rhinolophus ferrumequinum* (n  =  6, two males and four females) and *Myotis ricketti* (n  =  6, four males and two females) bats were captured while they were still in torpor from Fish Cave (30°20′N, 117°50′E), Anhui, China and Fangshan Cave (39°48′N, 115°42′E), Beijing, China, respectively. The temperatures in these caves ranged from 9 to 12°C, while the ambient temperature outside the caves was lower than 4°C. The captured *M. ricketti* (n = 3, two males and one female) and *R. ferrumequinum* (n  = 3, one male and two females) bats were sacrificed before arousal, when their core body and surface temperatures were 16 and 13°C, respectively. Their livers were rapidly removed and flash-frozen in liquid nitrogen. The remaining *M. ricketti* and *R. ferrumequinum* bats were sacrificed 48 hours after arousal to obtain liver tissues. Non-hibernating bats were sacrificed 48 hours after capture. The core body temperature of these bats was approximately 35°C at the time of sacrifice. The room temperature of the lab was maintained at 27°C. The snap-frozen tissue samples were stored in a −80°C freezer until used. Fresh pig livers were purchased from Fuxin Abattoir (Shanghai, China). Mice and rats were obtained from Sino-British Sippr/BK Lab Animal Ltd (Shanghai, China). All animals used for this study were sacrificed by cervical dislocation.

### Sample Preparation for Two-dimensional (2D) Gel Electrophoresis

Each (125 mg) of the bat liver samples was placed in a 2-ml tube containing ceramic beads (0.17 g, 1 mm in diameter) and homogenized in 1.7 ml of lysis buffer containing 1.5% Nonidet P-40 (Sigma, USA), 1x protease inhibitor (Roche, USA), and 1x nuclease mix (GE, UK) with a Precellys® 24 grinder (Bertin technologies, France) at 4°C. The cells in each homogenate were pelleted by centrifugation at 14,000 xg for 30 min, resuspended in 150 µl of rehydration buffer (7 M urea, 2 M thiourea, 2% CHAPS), and then sonicated at 100 w, 40 kHz for 20 minutes at 4°C to obtain soluble proteins. The proteins were divided into small (30 µl) aliquots and stored at −80°C until used. One aliquot of each sample was assayed for protein concentration by the Quick Start^™^ Bradford protein assay kit (Bio-Rad, USA) according to manufacturer’s instructions.

### 2D-Gel Electrophoresis

Electrophoresis of 2D gels was performed as previously described with slight modifications [32]. IPG strips (13 cm) with a linear pH gradient (pH 3–10) were loaded with protein samples by soaking each of them in 250 µl rehydration buffer containing 180 µg of protein for 20 hr at 16°C. The first-dimension isoelectric focusing (IEF) was performed with an Ettan IPGphore 3 (GE, UK) apparatus at 100 V for 6 h, 300 V for 6 h, 500 V for 3 h, 1000 V for 3 h, 5000 V for 1.5 h, 8000 V for 1.5 h, and finally at 8000 V for a total of 36,900 Vhr. After IEF, proteins on each strip were reduced and then separated by size using a Hofer SE600 Ruby electrophoresis apparatus (GE, UK) at 25 mA/gel. The 2D gels were stained with Coomassie brilliant blue G-250 [33]. Three independent runs of the samples from each pair (hibernation and active states) of bats of the same sex were performed; therefore, a total of 18 stained gels were obtained. Gel images were acquired with an Image Scanner III (GE, UK), and the amount of protein in each spot was calculated by using the ImageMaster^™^ 2D Platinum software version 7.0 (GE, UK). The volume of a protein spot was defined by the sum of the intensity pixel units within the spot. To adjust for quantitative variations in the intensity of protein spots, the spot volumes were normalized as percentages (%) of total volume of all the spots present in the gel. Statistical significance between spot volumes of hibernation and active groups was determined by one-way ANOVA (Holm-Sidak method). A *P* value <0.05 was considered significant.

### Protein Recovery

Recovery of proteins from each gel spot was performed as previously described [32]. Protein spots were manually picked from the 2D gels and destained with 25 mM ammonium bicarbonate. After 10 min dehydration with acetonitrile, the gel pieces were vacuum dried and rehydrated in 50 µl of 2% (v/v) 2-mercapteoethanol for 20 min at room temperature. The proteins in the gel pieces were then alkylated with 50% acetonitrile in 25 mM ammonium bicarbonate (pH 8.5) for 20 min at room temperature. Gel pieces were subsequently washed twice with 25 mM ammonium bicarbonate (pH 8.5) for 15 min, dehydrated with acetonitrile for 10 min, dried, and finally rehydrated and digested with 25 ng sequencing grade trypsin (Promega, Madison, WI) in 25 mM ammonium bicarbonate (pH 8.5) at 37°C for 16 h. After digestion, the tryptic peptides were extracted twice with 50% acetonitrile containing 0.1% formic acid for 15 min with moderate sonication and then dried under vacuum. The dried pellet was dissolved in 10 µl of 0.1% formic acid and then subjected to mass spectrometry (MS) using an LTQ-Orbitrap Discovery hybrid mass spectrometer with a nano-electrospray ion source (ThermoElectron, San Jose, CA) coupled to a nano-flow HPLC (Agilent Technologies 1200 series). The nano-flow HPLC system was coupled to a C18 PepMap 100 (5-mm length, 300-µm inner diameter, 5-µm based size) trap column. Peptides were eluted using a 13.5-cm long, 75-µm inner diameter tip column (YMC-Gel, Liquid Chromatography) with a 5–35% linear gradient of solution A (0.1% formic acid in water) and solution B (0.1% formic acid in acetonitrile) at a flow rate of 0.5 µl/min for the first 30 min and with 95% solution B for the next 10 min.

### Protein Identification and Construction of Bat Protein Database

The MS spectra (survey scan) of peptides were acquired at high resolution (M/δM, 60,000 full width half maximum) within the m/z range of 200–2000, and a series of precursor ions were selected for the MS/MS scan. The resulting RAW data were processed by the Xcalibur 2.0.7 SR1 software package. An in-house program was used to convert the MS/MS information to DTA files. All DTA files were analyzed by the software TurboSequest version 27 (rev. 11) to search for the best matched peptides from the bat protein sequence database with 37,581 entries specially constructed for this study. This non-redundant bat protein database contained bat peptide sequences (FASTA files) downloaded from Ensemble, including sequence packages of *Myotis lucifugus* (v.2.0.68) and *Pteropus vampyrus* (v.1.68). The identified proteins were then compared with those in the UniprotKB database (Swiss-Prot, 2012/09) with 53,6789 entries in order to obtain their Uniprot accession numbers. Only peptides resulted from tryptic cleavages were analyzed, and the maximum number of missed cleavage sites was set to 2. Oxidative forms of methionines were allowed. The mass tolerance for precursor peptide ions was set to 3.5, and the fragment ion tolerance was set to 1. TurboSequest results were filtered by the criteria similar to those of Qian *et al* [34], and all accepted results had a DelCN (delta Cn) of 0.1 or greater indicating a high confidence in TurboSequest results [34]. A protein was identified when at least two unique peptides were matched with the Xcorr score of each peptide higher than 2.5.

### Ingenuity Pathways Analysis

Accession numbers and expression changes of the proteins listed in Table 1 were uploaded as an Excel file into the Ingenuity Pathway Analysis (IPA) software (Ingenuity® System) for grouping of functional networks of the proteins according to their functions and interactions. The significance (*P* value of overlap) was calculated by the Fisher’s exact test. Both upregulated and downregulated proteins listed in Table 1 were analyzed for their possible upstream transcriptional regulators.

**Table 1:** Comparison of protein expression levels in hibernation and active states of *Myotis ricketti*.

ID^a^	Accession number^b^	Protein Identification (s)	MW/pI^c^	MPN^d^	PC^e^ (%)	FC^f^(H/A)
1	Q6FG96	60S ribosomal protein P2, RPLP2 protein^g^	11657.85/4.38	9	48.70	-
2	Q6FG96	60S ribosomal protein P2, RPLP2 protein^g^	11657.85/4.38	9	48.70	↓1.25
3	P02608	Myosin regulatory light chain 2, skeletal muscle isoform type 2	18895.34/4.79	24	99.00	↓1.45*
4	P60661	Myosin, light polypeptide 6	16798.86/4.56	7	20.53	↓1.29
5	P67936	Tropomyosin alpha-4 chain	28390.62/4.67	77	99.00	↓1.72**
6	P53505	Actin, cytoplasmic type 5^g^	41849.89/5.30	14	33.43	↓1.28
7	P53505	Actin, cytoplasmic type 5^g^	41849.89/5.30	14	33.43	↓1.13
8	P53505	Actin, cytoplasmic type 5^g^	41849.89/5.30	14	33.43	-
9	P81947	Tubulin alpha-1B chain	50151.63/4.49	60	59.56	↑2.20*
10	P02548	Neurofilament light polypeptide	62514.51/4.58	9	35.48	-
11	P55072	Transitional endoplasmic reticulum ATPase	89190.61/5.14	26	31.89	-
12	P38646	Stress-70 protein, mitochondrial	68759.00/5.44	201	86.78	↑3.65**
13	P10809	60 kDa heat shock protein, mitochondrial^g^	57962.86/5.24	37	49.04	-
14	P10809	60 kDa heat shock protein, mitochondrial^g^	57962.86/5.24	54	20.93	-
15	P02680	Fibrinogen gamma chain	50632.74/5.62	8	20.37	-
16	P14639 http://www.uniprot.org/uniprot/P14639	Serum albumin	69188.00/5.80	24	24.34	↓1.11
17	Q8C196	Carbamoyl-phosphate synthase [ammonia], mitochondrial^g^	160307.8/6.09	383	88.05	↑1.50
18	Q8C196	Carbamoyl-phosphate synthase [ammonia], mitochondrial^g^	160307.8/6.09	383	88.85	↑1.43*
19	Q8C196	Carbamoyl-phosphate synthase [ammonia], mitochondrial^g^	160307.8/6.09	383	88.85	↑1.11
20	P05165	Propionyl-CoA carboxylase alpha chain, mitochondrial^g^	74246.99/6.16	25	29.76	-
21	P05165	Propionyl-CoA carboxylase alpha chain, mitochondrial^g^	74246.99/6.16	29	33.84	-
22	P05165	Propionyl-CoA carboxylase alpha chain, mitochondrial^g^	74246.99/6.16	29	33.84	-
23	P05165	Propionyl-CoA carboxylase alpha chain, mitochondrial^g^	74246.99/6.16	29	33.84	↓1.25
24	Q9ET01	Glycogen phosphorylase, liver form^g^	97331.88/6.65	25	22.57	↓1.29
25	Q9ET01	Glycogen phosphorylase, liver form^g^	97331.88/6.65	26	31.65	↑1.45*
26	Q9ET01	Glycogen phosphorylase, liver form^g^	97331.88/6.65	26	31.65	↓1.29
27	Q9ET01	Glycogen phosphorylase, liver form^g^	97331.88/6.65	39	45.27	-
28	Q9ET01	Glycogen phosphorylase, liver form^g^	97331.88/6.65	81	66.41	-
29	Q9ET01	Glycogen phosphorylase, liver form^g^	97331.88/6.65	39	45.27	↓2.38
30	Q5MIB5	Glycogen phosphorylase, liver form^g^	97331.88/6.65	81	66.41	↓1.78
31	Q8K0E8	Fibrinogen beta chain	54752.71/6.68	44	51.35	↓1.31
	P11598	Protein disulfide-isomerase A3	56623.37/5.88	18	32.28	↓1.31
32	P00439	Phenylalanine-4-hydroxylase	51862.09/6.15	8	14.73	↑1.89**
33	P49410	Elongation factor Tu, mitochondrial	45049.51/6.20	4	09.07	↑1.15
34	Q3SZJ0	Argininosuccinate lyase	52743.07/6.04	13	20.26	-
35	P54868-2	Hydroxymethylglutaryl-CoA synthase, mitochondrial^g^	52383.34/ 6.64	38	45.14	-
36	P54868-2	Hydroxymethylglutaryl-CoA synthase, mitochondrial^g^	52383.34/ 6.64	21	38.52	-
37	P54868-2	Hydroxymethylglutaryl-CoA synthase, mitochondrial^g^	56849.61/8.86	24	35.60	-
38	Q64I00	Glutamate dehydrogenase 2, mitochondrial^g^	61385.85/8.63	124	99.00	↓1.28*
39 40	Q64I00 Q3SZB4	Glutamate dehydrogenase2, mitochondrial^g^ Medium-chain specific acyl-CoA dehydrogenase, mitochondrial	61385.85/8.63 43586.83/7.02	212 8	99.00 16.14	↓1.11* ↑1.19
41	Q5EA20	4-hydroxyphenylpyruvate dioxygenase	44832.04/6.25	26	48.22	↑1.47**
42	P25708	NADH dehydrogenase [ubiquinone] flavoprotein 1, mitochondrial	48499.21/7.08	38	40.30	-
	O09173	Homogentisate 1,2-dioxygenase^g^	49959.92/6.85	9	11.91	-
43	O09173	Homogentisate 1,2-dioxygenase^g^	49959.92/6.85	19	30.34	↑3.35**
44	Q8CI38	Homogentisate 1, 2-dioxygenase^g^	49900.53/6.85	9	09.66	↑1.21
45	P05089	Arginase-1	35664.07/6.72	14	99.00	-
46	A5PKH3	Fumarylacetoacetase^g^	46024.69/6.49	20	25.30	-
47	A5PKH3	Fumarylacetoacetase^g^	46024.69/6.49	22	33.17	↑1.69
48	P11708	Malate dehydrogenase, cytoplasmic	36299.97/6.15	28	52.56	-
49	Q5FZI9	Uricase	34942.79/8.22	78	84.21	-
50	Q3T165	Prohibitin	29804.10/5.57	125	99.00	↑1.18
51	G3HAE3	ATP synthase subunit alpha, mitochondrial	31185.66/6.92	72	42.68	-
52	Q76I81	40S ribosomal protein S12	14383.74/7.01	10	41.61	↑1.14
53	P11757	Hemoglobin subunit alpha	15139.41/7.98	45	99.00	↓1.30*

a ID: spot number of proteins indicated in figures 1, S1, and S2.

b Accession number: Uniprot ID number of each protein identified.

c MW/pI: Theoretical molecular weight and pI calculated by the Compute tool hosted in ExPASy.

d MPN (matched peptide number): Number of unique peptides matched to the protein by TurboSequest search.

e PC (peptide coverage, %): the combined length (number of amino acids) of matched peptides of the protein divided by the full length (number of amino acids) of the protein.

f FC (fold change, H/A): The protein amount in hibernation (H) state was compared to that in active (A) state. ↑, up-regulation; ↓, down-regulation; –, no significant change. All fold changes were statistically significant with a *P* value <0.05; those marked with asterisks had a smaller *P* value: *, *P*<0.01; **, *P*<0.001.

g Multiple spots: potential existence of post-translational modification forms of the same protein.

### Western Blot Validation

To investigate enzyme evolution, liver proteins were extracted from hibernators (*M. ricketti* and *R. ferrumequinum*) and non-hibernators (*Cynopterus sphinx*) of bats as well as pigs, mice, and rats. Proteins (20 µg/lane) in each sample were separated by 12.5% SDS-PAGE and then transferred onto a PVDF membrane. A reversible Ponceau staining of the membrane was done before blocking to visualize protein bands and to ensure that proteins were successfully transferred to the membrane [35]. The protein-bearing PVDF membranes were blocked for 1 h in blocking solution (5% skim milk and 1% BSA) and then reacted with a series of primary antibodies (anti-PAH, 1∶500; anti-HPD, 1∶500; anti-HGD, 1∶1000; anti-FAH, 1∶250; anti-CPS1, 1∶500; anti-GLUD1/2, 1∶500) in TBS-T. Anti-PAH (sc-15112), anti-FAH (sc-67288), anti-HPD (sc-98596), anti-CPS1 (sc-10516), and anti-GLUD1/2 (sc-160382) antibodies were purchased from Santa Cruz Biotechnology, Inc. Anti-HGD (D01P) was obtained from Abnova Corporation. These antibodies were chosen because of their ability to recognize conserved epitopes of the target proteins of many mammalian species. After washing, the blots were treated with secondary antibody and visualized according to manufacturer’s instructions (Santa Cruz Biotechnology, Santa Cruz, CA). Images were acquired with the ImageQuant^™^ LAS-4000 (Amersham Biosciences), and band intensities were quantified with the ImageQuant^™^TL software (version 7.0, Amersham Biosciences). The intensity of each band was normalized to that of the corresponding Ponceau-stained protein band in the same lane. The results expressed as mean ± SD were calculated from six repeats and analyzed by one-way ANOVA. A *P* value <0.05 was considered significant.

### Determination of Phenylalanine and Acetoacetate levels in liver tissues

Determination of phenylalanine and acetoacetate levels were performed according to the instruction manuals of EnzyChrom^™^ phenylalanine (EPHE-100) and ketone body (EKBD-100) assay kits (BioAssay Systems, Hayward, USA). Briefly, liver tissue (20 mg) was homogenized in 200 µl PBS (137 mM NaCl, 2.7 mM KCl, and 10 mM Na_2_HPO_4_). The cell debris was then removed by centrifugation, and the clarified supernatant was assayed for phenylalanine and acetoacetate levels on a flat-bottom 96-well microplate. To synchronize all reactions, the ice-cold assay reagents were added to the plate chilled on ice. The microplate was then incubated at room temperature for 5 minutes for acetoacetate reactions and 20 minutes for phenylalanine reactions. In the phenylalanine assay, phenylalanine was oxidized by phenylalanine dehydrogenase in the presence of NAD^+^ which was converted to NADH. The latter reduced a propriety dye to a highly fluorescent product. Phenylalanine concentration in a sample was determined by comparing the resulting fluorescence intensity (λ_exc/em_  = 530/595 nm) against those of a standard curve of known concentrations of phenylalanine. For acetoacetate assay, 3-hydroxybutyrate dehydrogenase converted acetoacetate and NADH to 3-hydroxybutyrate and NAD^+^ at pH 7.0. The decrease in NADH levels was determined by measuring the absorbance at 340 nm. Acetoacetate concentration in a sample was determined by comparing the OD_340_ value against those of a standard curve of known concentrations of acetoacetate. The results were analyzed by one-way ANOVA, and a *P* value <0.05 was considered significant.

### Molecular Cloning and Evolutionary Analysis

The coding regions of *PAH*, *HPD*, *HGD*, and *FAH* genes of 12 different bat species (Table S1) were cloned. These bat species were used because whether they hibernate is known [30], [36]. Total RNAs were isolated from liver tissues using the RNAiso Plus kit (TaKaRa). RT-PCRs were then performed using the primers shown in Table S2. The PCR products were purified and cloned into pGEM-T Easy vectors (Promega). Correct recombinant clones were sequenced from both directions of the inserts using ABI 3730 sequencer (Applied Biosystems). All new sequences obtained in this study were submitted to the GenBank. The accession numbers of these sequences and those downloaded from Ensemble are shown in Table S1. The nucleotide sequences of the four enzymes were aligned using ClustalW [37], [38]. To determine the selective pressure acting on the genes, the pairwise ω value, i.e., d_N_/d_S_ ratio (non-synonymous substitution rate/synonymous substitution rate) of each gene, was calculated using the software Swaap version 1.0.3 [39], [40].

### Structure Based Sequence Alignment and Homology Modeling

The amino acid sequences of bat PAH, HPD, HGD, and FAH were deduced from their nucleotide sequences. To identify the amino acids of these enzymes that were conserved in hibernators but diverged in non-hibernators of bats and to correlate enzyme functions with their structures, structure-based sequence alignments were performed using the 3D-Expresso software [41]. The templates for the alignment were the amino acid sequences of enzymes from *Homo sapiens* (human), *Rattus norvegicus* (rat), or *Mus musculus* (mouse) with known structures (Table S3). Since the alignment results showed greater than 96% similarity between these four enzymes of bats and each of their corresponding template, the amino acid sequences of PAH, HGD, HPD, and FAH of *Myotis ricketti* were aligned separately with each of their structural template using Deepview-Swiss-PdbViewer to obtain the three-dimensional structures of the enzymes [42]. The files generated were exported into SWISS-MODEL for homology modeling [43]. The Discovery Studio Visualizer version 3.1 (Accelrys®, San Diego, CA) was used to display the 3D structures. Databases PAHdb, HGMD®, and UniProtKB (Swiss-Prot) were utilized to correlate the conserved residues in the enzymes with their enzymatic activities.

## Results

### Liver Proteome of *Myotis ricketti*

Expression profiles of liver proteins in hibernating (torpid) and non-hibernating (active) states of *M. ricketti* were compared by proteomic analyses (Figures 1A and S1), and the expression of a number of proteins was found to be increased in torpid bats (Table 1). PAH, the rate-limiting enzyme catalyzing the conversion of phenylalanine to tyrosine, showed a 1.89-fold increase in expression (Figure 1B). HPD, the second enzyme in tyrosine catabolism, had a 1.47-fold increase in expression (Figure 1B). One isoform of HGD, that cleaves the aromatic ring during tyrosine degradation, was increased by 3.35 fold (Figures 1A and 1B). One isoform of FAH, the last enzyme in tyrosine catabolism, also had a significant increase (1.69 fold) in expression (Figure 1B).

**Figure 1:**
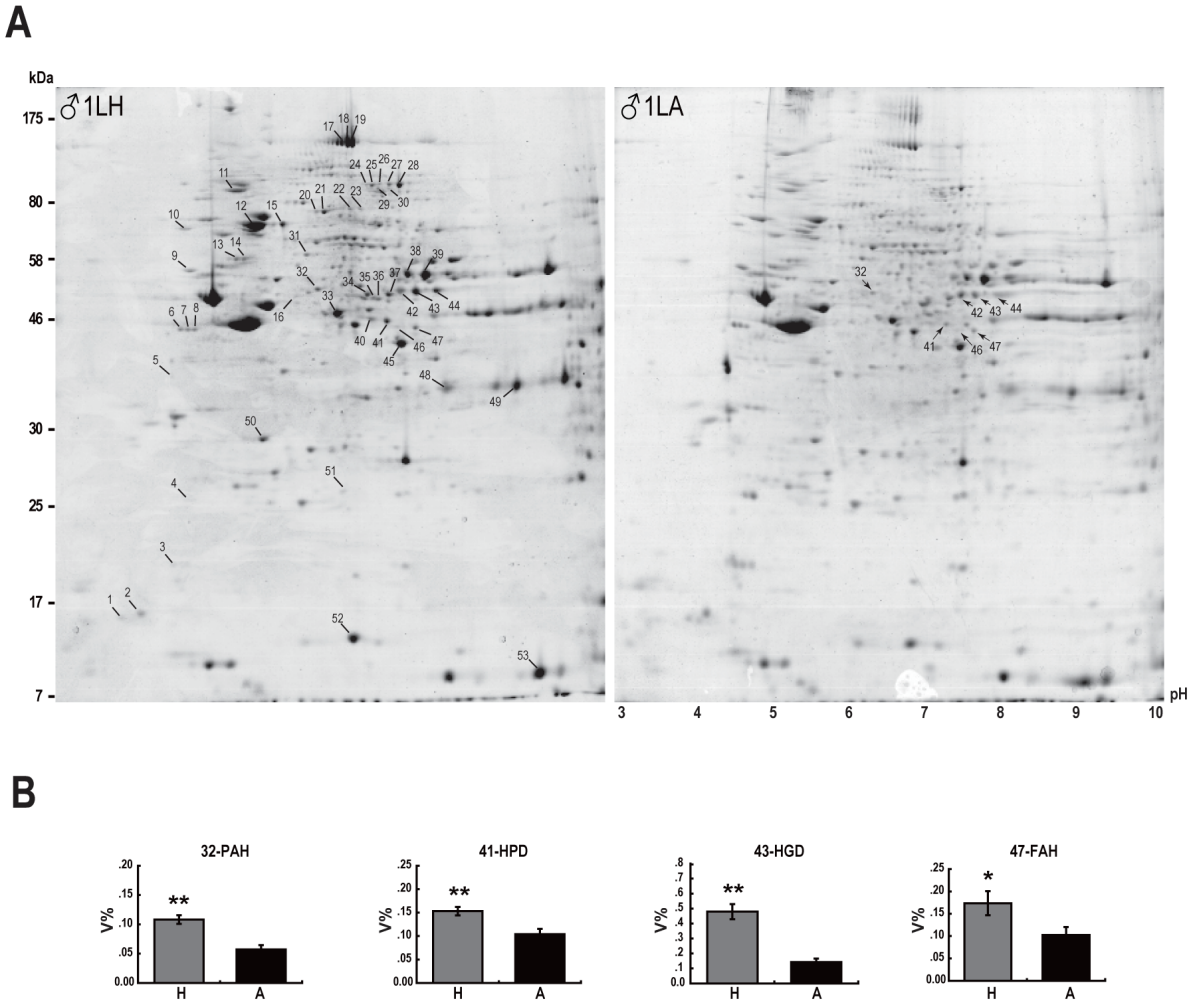
Two-dimensional (2D) gel electrophoresis of liver proteins of *Myotis ricketti*. (**A**) The 2D gels of the first pair of male bats in hibernation (left panel, 1LH) and active states (right panel, 1LA) are shown. The numbers on the left of the gel indicate the positions and approximate molecular mass (in kDa). The numbers below the gel denote the approximate pH gradient across the gel. Protein spots identified are labeled with ID numbers shown in Table 1. (**B**) Expression levels of PAH, HPD, HGD, and FAH were determined by the ImageMaster^™^2D Platinum software. V% indicates spot volume normalized as percentages of the total volume of all spots in the gel. The average levels of each protein in hibernation (H) and active (A) states are represented by light-gray and black bars, respectively. Each bar represents results of nine gels. Experimental data are mean ± SD. A *P* value <0.05 (one-way ANOVA test) is considered significant: *P<0.05 and **P<0.001. (The 2D gels of other bat pairs are shown in Figure S1.)

Changes in the levels of several other proteins involved in nitrogen metabolism were also observed. For example, the expression of glutamate dehydrogenase 2 (GLUD2) that catalyzes glutamate deamination to generate α-ketoglutarate and ammonia was slightly decreased, and the carbamoyl-phosphate synthase (CPS1), a rate-limiting enzyme involved in urea production for metabolic removal of ammonia (NH_3_), was found to be significantly increased in expression during hibernation (Table 1 and Figure S2).

The levels of some structural proteins in the liver, such as myosin regulatory light chain 2 (MRLC2), myosin light chain peptide 6 (MYL6), tropomyosin alpha-4 chain (TPM4), fibrinogen beta chain (FGB), and two isoforms of cytoplasmic actin (ACTG5) were decreased, but the amount of tubulin alpha-1B (TUBA1B) was increased (Table 1). Moreover, the chaperonin mtHSP70 was found to be highly expressed. Increased expressions of the mitochondrial elongation factor Tu (TUFM) and the 40S ribosomal protein S12 (RPS12) were also observed. However, the expression of one isoform of the 60S acidic ribosomal protein P2 (RPLP2) was decreased. A decreased expression of the hemoglobin subunit alpha was also seen.

Many enzymes related to glucose production were also differentially expressed. Four of seven glycogen phosphorylase isoforms (Spot ID 24–30) that are rate-limiting enzymes for glucose synthesis were decreased in expression in torpid bats (Table 1). The expression level of one isoform of propionyl-CoA carboxylase (PCCA) that converts propionyl-CoA to methylmalonyl-CoA was decreased. Overexpression of the medium-chain specific acyl-CoA dehydrogenase (ACADM) that is involved in fatty acid beta-oxidation was also observed.

**Figure 2:**
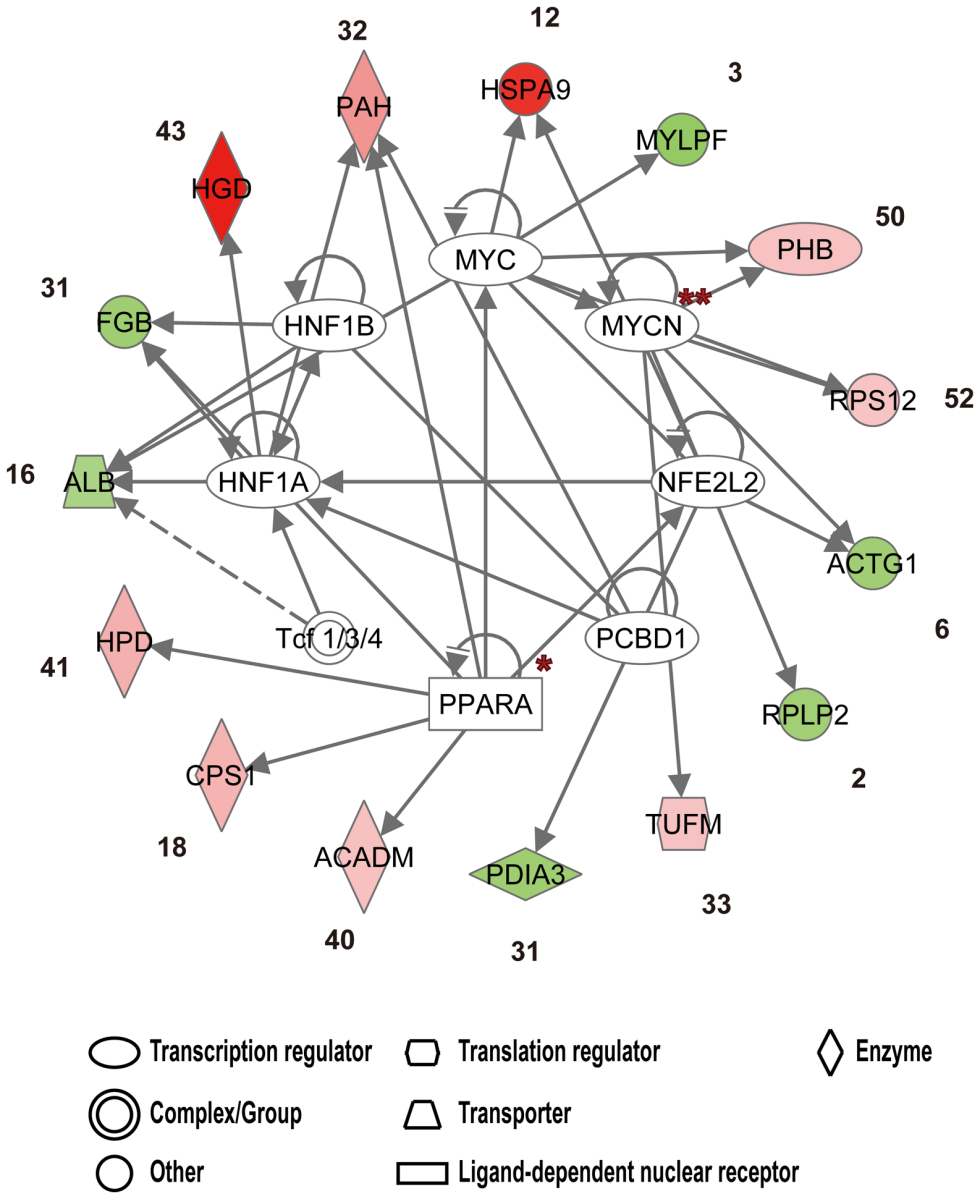
IPA analyses of transcription regulators. The interaction between transcription factors (inner circle) and both upregulated and downregulated proteins listed in Table 1 (outer circle) is shown. Upregulated and down-regulated expressions are indicated in red and green, respectively, the darker the red color the higher level of protein expression. Numbers indicate the protein IDs shown in Table 1. Arrows denote activation, and T-bars represent inhibition. Solid and dashed arrows represent direct and indirect interactions, respectively. All *P* values of overlap are <10^−3^. Asterisks * and ** indicate *P* values of overlap <10^−5^ and <10^−6^, respectively.

To obtain an insight into the regulation of these differentially expressed proteins, a knowledge based software IPA (Ingenuity Pathways Analysis) was used to analyze the data (Figure S3). Results of IPA analyses of upregulated and downregulated proteins listed in Table 1 showed that some transcription factors, such as peroxisome proliferator-activated receptor (PPARA), MYC proteins, hepatocyte nuclear factors (HNF1A and HNF1B), and nuclear factor (erythroid-derived 2)-like 2 (NFE2L2) were highly associated with bat hibernation (Figure 2).

### Western Blotting of Enzymes in the Phe and Tyr Catabolic Pathway

**Figure 3:**
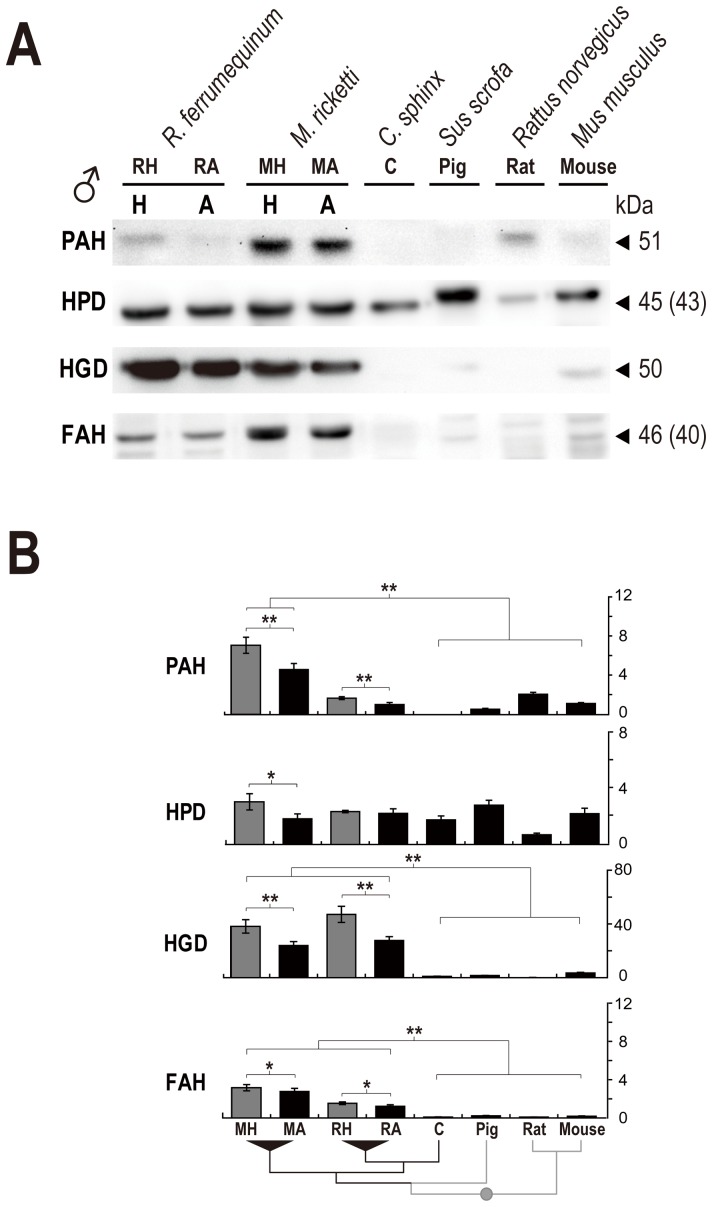
Expression patterns of the enzymes responsible for the catabolism of Phe and Tyr. Protein expressions of PAH, HPD, HGD, and FAH in the livers of pigs, rats, mice, and male (♂, **A**) bats were determined by Western blotting. H and A indicate bats in hibernation and active states, respectively. Arrow heads indicate predicted molecular weight (kDa) of the proteins; the numbers in parentheses denote observed molecular weights. (**B**) Relative protein levels (y-axis) are represented as mean ± SD. The lowest level of a detectable protein is considered as 1. Hibernation (H) and active (A) states are represented by light-gray and black bars, respectively. Phylogenetic trees are drawn at the bottom of the panel. Statistical significance: *P<0.05, **P<0.001.

The proteomic results suggested that PAH, HPD, HGD, and FAH were co-upregulated during hibernation of *M. ricketti* bats (Figure 1). To confirm these results, Western blot analyses were performed. The results showed that PAH, HPD, HGD, and FAH in the liver were upregulated for 1.54, 1.66, 1.37, and 1.14 fold, respectively, in torpid *M. ricketti* bats (Figure 3B). To investigate the evolution of these enzymes, their expressions in the livers of other bats and several mammalian species (pig, rat, and mouse) were examined. In another bat hibernator *R. ferrumequinum*, the expression levels of PAH, HGD, and FAH were increased by 1.67, 1.47, and 1.26 fold, respectively, but those of HPD had no change (Figure 3B). The molecular weights of detected HPD and FAH were 43 kDa and 40 kDa, respectively, slightly lower than the predicted molecular weights (Figure 3A) [44], [45]. The expression levels of PAH, HPD, HGD, and FAH of both hibernating species of bats were higher than those of the non-hibernating *C. sphinx* bats (Figure 3). The co-upregulation of PAH, HGD, and FAH in different suborders of hibernating bats (i.e., *M. ricketti* in Yangochiroptera and *R. ferrumequinum* in Yinpterochiroptera) suggests an essential role of these enzymes during hibernation. In the hibernating *M. ricketti* and *R. ferrumequinum* bats, both HGD and FAH were expressed at higher levels than in those of non-hibernating mammalian species (pig, rat, and mouse) regardless of their torpid or active states and gender (Figures 3 and S4).

### Determination of Phenylalanine and Acetoacetate levels in the liver

To correlate the increase in phenylalanine catabolizing enzymes with phenylalanine breakdown, the concentrations of phenylalanine and acetoacetate in the livers of *M. ricketti* and *R. ferrumequinum* bats in hibernation and in active states were compared. The results were also compared to those of non-hibernating *C. sphinx* bats and two species of mammals, pig and mouse (Table 2). The average liver phenylalanine level of *R. ferrumequinum* bats (n  =  4) during the active state was 221.75±34.70 µM. This concentration was reduced to 127.59±16.90 µM (a 40% decrease) in hibernation. In contrast, liver acetoacetate levels were increased from 0.75±0.05 mM in active state to 0.83±0.06 mM (a 10% increase) in hibernation state. Since acetoacetate is a catabolite of phenylalanine, these results suggest that the catabolism of phenylalanine is more active in *R. ferrumequinum* bats during hibernation. A more dramatic decrease (from 268.11±49.09 µM to 52.43±25.45 µM, an 80% decrease) in phenylalanine levels and increase (from 0.30±0.09 mM to 0.72±0.17 mM, a 60% increase) in acetoacetate levels in active than in hibernation state were observed in *M. ricketti* bats. Liver phenylalanine levels in *C. sphinx* bats (284.80±48.54 µM), pigs (316.97±35.59 µM), and mice (244.44±50.46 µM) were similar to those of *R. ferrumequinum* and *M. ricketti* bats in active state. Liver acetoacetate levels were 0.25±0.13 mM in *C. sphinx* bats and were 0.18±0.06 mM in mice, but were lower than the detection limit of the assay kit in pigs (Table 2).

**Table 2:** Concentrations of Phenylalanine and Acetoacetate in the liver.

Animal group^a^	Phenylalanine (µM)^c^	Acetoacetate (mM)^c^
***R. ferrumequinum*** ** (H)** b	127.59±16.90	0.83±0.06^d^
***R. ferrumequinum*** ** (A)** b	221.75±34.70^d^	0.75±0.05
***M. ricketti*** ** (H)** b	52.43±25.45	0.72±0.17^d^
***M. ricketti*** ** (A)** b	268.11±49.09^d^	0.30±0.09
***C. sphinx***	284.80±48.54	0.25±0.13
***R. norvegicus*** ** (pig)**	316.97±35.59	—e
***M. musculus*** ** (mouse)**	244.44±50.46	0.18±0.06

a Four (n = 4) animals in each group were used.

b Bats were sacrificed at hibernation (H) or 48 h after arousal (A).

c Concentrations determined were represented as mean ± SD of three repeats.

d Statistical significance (*P* <0.05) was found between hibernation and arousal states of bats.

e The concentration was below the detection limit of the assay kit.

### Sequence Analyses of Bat Enzymes

**Figure 4:**
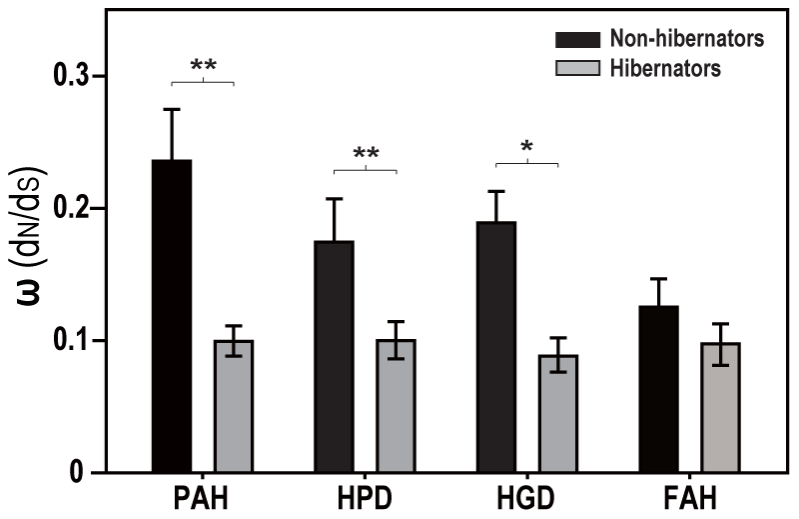
Selective pressure on *PAH*, *HPD*, *HGD*, and *FAH*. The selective pressure (ω value) of each gene was determined by comparing the ratio of synonymous to nonsynonymous nucleotide changes of each protein from several species of hibernators (gray bars) and non-hibernators (black bars) of bats. Data are presented as mean ± SD. Statistical significance was determined by one-way ANOVA: *P<0.05, **P<0.001.

To study the evolution of these catabolic enzymes in adaptation to hibernation, genes encoding PAH, HPD, HGD, and FAH of several hibernating and non-hibernating species of bats were cloned and sequenced. Amino acid sequence alignments of these proteins are shown in Figures S5 and S6. The bat sequences were divided into hibernation and non-hibernation datasets to compare the differences in selective pressure. Results showed that all the proteins examined were under a strong purifying selection with an ω (d_N_/d_S_) value less than 0.3 (Figure 4). Three enzymes (PAH, HPD, and HGD) displayed a stronger selective constraint in hibernators than in non-hibernators (Figure 4).

### Comparison of Enzyme Features in Hibernating and Non-hibernating Bats

**Figure 5:**
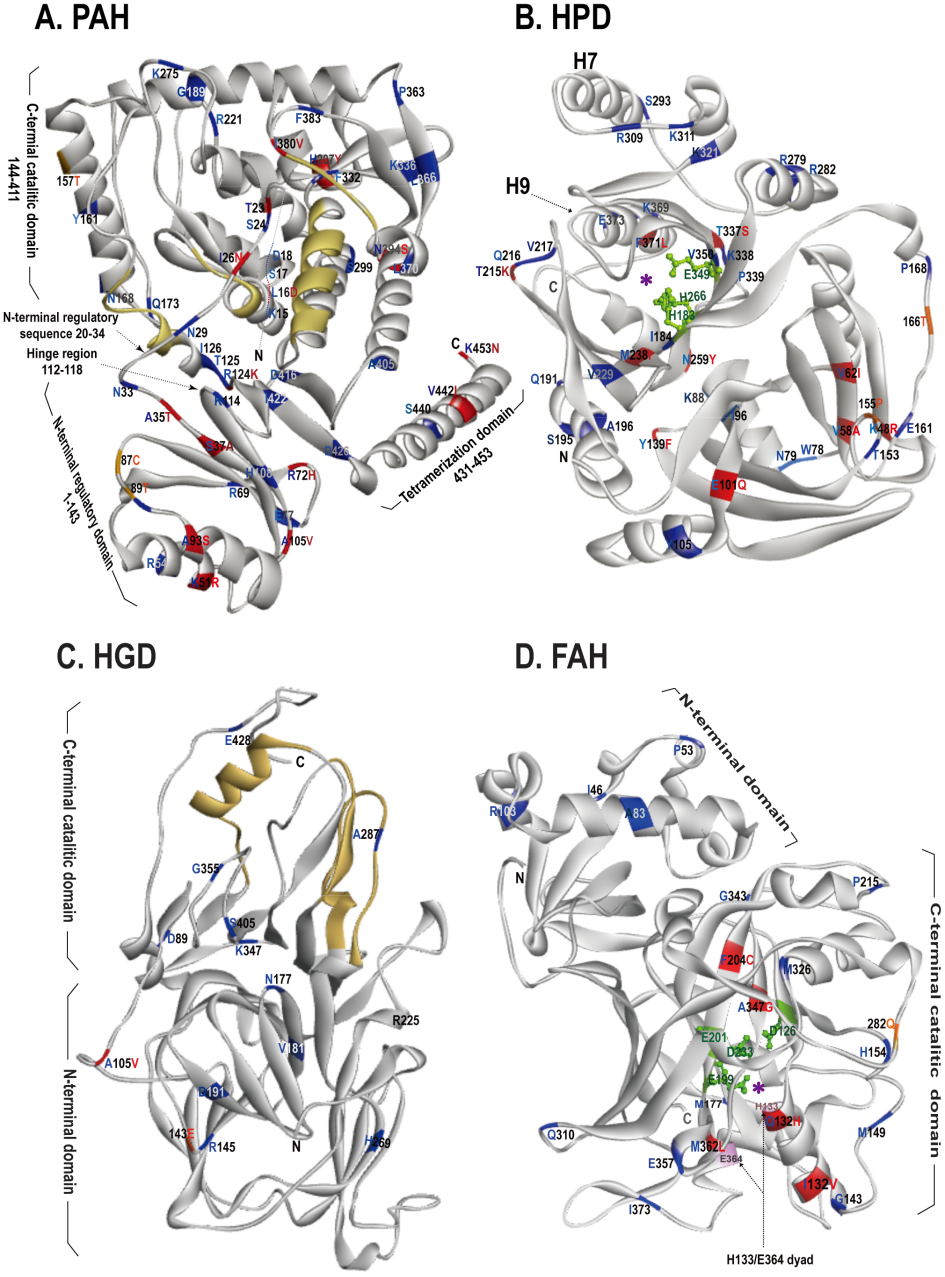
Stereoview of PAH, HPD, HGD, and FAH. Amino acid residues that are conserved in all hibernators of bats but are different or diverged among non-hibernators of bats are indicated in red or blue, respectively. Orange color denotes positions that are conserved in all non-hibernating bats but diverged among hibernating bats. N-terminus and C-terminus are labeled as N and C, respectively. (**A**) The catalytic domain of PAH consisting of residues R131-L137, L250-R253, D316-W327, and E377-I380 is colored in yellow. The autoregulatory region is located between G20 and G34 at the N-terminus. Residues R112-T118 located in the hinge region are essential for phenylalanine-modulated proteolytic cleavage, and residues L431-K453 are required for the formation of a fully active enzyme tetramer. (**B**) Activity cave of HPD is indicated with a purple asterisk. Green color denotes residues and side chains of H183, H266, and E349 for binding metal ions and water molecule. (**C**) The active site of HGD composed of residues F282-T299, P320-K327, and M368-K385 is colored in yellow. Residue H269 is critical for trimerization of HGD and activation of the enzyme. (**D**) The purple asterisk denotes the activity cave of FAH. Green color indicates residues and side chains of D126, E199, E201, and D233 for Ca^2+^ binding. The H133/E364 dyad is critical for the catabolism of enzyme substrate.

To correlate structures to functions, the amino acid sequences of PAH, HPD, HGD, and FAH from hibernating and non-hibernating bats and their structure templates from human (PAH, HPD, HGD, and FAH), rat (PAH and HPD), and mouse (FAH) were analyzed by structure-based sequence alignments (Figures S5, S6, and Table S3). Results showed an average of 98% similarity among bat PAH and its template sequences (Figure S5A). A total of 50 PAH amino acids were found to be 100% conserved in all seven bat hibernators but were variable in three non-hibernators (Figure 5A). Among these, twenty-five positions (S17, T23, R54, R69, R72, E77, P90, A93, A105, H108, R124, T125, Y161, N168, Q173, G189, H207, K275, F332, P363, L370, I380, F383, D416, and I422; where the letter indicates amino acid residue and the number denotes position) had been shown to undergo missense or nonsense mutations leading to phenylketonuria (PKU) in humans [46], [47]. Thirteen of these (K15, L16, S17, D18, T23, S24, I26, N29, K114, E377, I380, V442, and K453) were located in the regions that regulate enzyme activities [17]. Four positions including K15, L16, S17, and D18 were situated at the N-terminus between S2 and Q21, which stabilizes the secondary structure of PAH in its phosphorylated form [17]. Three positions T23, I26, and N29 were located in the autoregulatory segment (G20–G34) that is predicted to move away to allow entry of phenylalanine when PAH is activated by phosphorylation at S17 (Figure 5A) [17], [18]. Residue K114 was located in a hinge region (R112–T118) that mediates phenylalanine-modulated proteolytic cleavage (Figure 5A). Residues E377 and I380 were in the last segments of the active site of the catalytic domain encompassing R131-L137, L250-R253, D316-W327, and E377-I380 (Figure S5A) [17], [18]. Residues V442 and K453 were located in the C-terminal coiled-coil domain which mediates formation of a fully active enzyme tetramer *in vivo* (Figure 5A). Residue K453 had been found to be deleted in some human patients with PKU (Figure S5A) [48].

For HPD sequences, there was a 96% similarity among those of bats and their templates (Figures 5B and S5B). Thirty-six HPD amino acid positions were conserved in all hibernators but were diverged among the non-hibernators of bats. Five residues N79, K88, 166T, P168, and N259 were located in the domain associated with the interface of the dimeric HPD, and positions I96 and Y139 were in the core of a highly stable region of the enzyme (Figure 5B) [19], [46]. Residues I184, V229, M238, and V350 were located in the center of an open twisted barrel-like beta sheet that forms the active site with a mental ion (iron or cobalt) held by H183, H266, and E349 (Figure S5B) [19]. A cluster of hydrogen bonds were present near the C-terminus and the active site consisting of Y295 (from helix H7), Q334, E349, and N363 (from helix H9) [19]; therefore, amino acid substitutions at S293 of helix H7 or K369, F371, and E373 of helix H9 may affect packing or stability of the enzyme.

Alignment of HGD sequences from humans and hibernators and non-hibernators of bats revealed a similarity of 98%. A total of 12 positions were conserved in hibernators but were variable in non-hibernators (Figures 5C and S6A); four of which (R145, V181, H269, and A287) had been reported to have missense or nonsense mutations leading to alkaptonuria (AKU) in humans [46]. Residue E428 was found to be deleted in some AKU patients [46]. The Cβ and Cγ atoms of H269 had been shown to interact with the side chain of R225 located in the intersubunit interface of the hexameric HGD through van der Waal’s contacts (Figure 5C) [20]. Residue A287 was located in the active site (F282-T299, P320-K327, and M368-K385) of the enzyme (Figure 5C) [20]. Since the activity of HGD is dependent on its hexameric quaternary structure, any amino acid substitutions that may affect electrostatic or hydrophobic contacts among the HGD subunits are unfavorable [20], [21].

Structure based sequence alignment of the metalloenzyme FAH among hibernators and non-hibernators of bats and mouse demonstrated a greater than 98% similarity. Nineteen conserved positions were found (Figure S6B). Missense and nonsense mutations had been found at positions G343 and E357, respectively, causing the most severe tyrosine metabolic disease in humans, namely type 1 tyrosinemia [23] (Figure 5D). More conserved residues were found in the C-terminal domain of FAH, including M149, H154, and M177 located in the inter surface of the FAH dimer, and Q132, I139, and M362 located in the helices that form the catalytic pocket [22]. Variations in sequences were seen at F204, G343, and A347 in the mixed C-terminal beta-roll which is critical for Ca^2+^ and substrate binding (Figures 5D and S6B) [22], [24].

## Discussion

Some mammalian hibernators suffer only minor loss of skeletal or liver proteins despite the lack of food intake during hibernation [5], [11], [12]. Our observation of increased expressions of mitochondrial stress-70 protein, prohibitin, 40S ribosomal protein, and mitochondrial elongation factor Tu (Table 1) is consistent with the postulation of continual protein synthesis and maintenance of protein folding during hibernation. However, contrary to the hypothesis of amino acid preservation, we found evidence which suggests amino acid catabolism as four enzymes (PAH, HPD, HGD, and FAH) involved in phenylalanine and tyrosine catabolism were co-upregulated in the liver of torpid *Myotis ricketti* bats (Figures 1 and S1). We also showed that the levels of phenylalanine in the liver were significantly decreased (Table 2), and those of acetoacetate were decreased in these bats (Table 2). Since acetoacetate is a catabolite of phenylalanine, these findings demonstrate that phenylalanine and tyrosine metabolism is more active in torpid than in active states of hibernating bats.

Because PAH converts phenylalanine to tyrosine, these results suggest that tyrosine is produced in order to maintain proper levels of neurotransmitters (e.g., catecholamines) or hormones (e.g., noradrenaline and thyroid hormones) to mediate thermogenesis and control energy balance during hibernation [15], [16]. Since excessive accumulation of tyrosine and its catabolites (e.g., 4-hydroxyphenylpyruvate and homogentisate) is highly toxic [23], they need to be removed. It is likely that overexpression of HPD, HGD, and FAH during hibernation is also a mechanism of detoxification as HPD converts 4-hydroxyphenylpyruvate to homogentisate, and HGD converts homogentisate to meleylacetoacetate which is then metabolized by FAH to become fumarate and acetoacetate. Fumarate is an intermediate in the tricarboxylic acid cycle in which energy (ATP) is produced, and acetoacetate can be reduced to β-hydroxybutyrate in the liver. Acetoacetate, β-hydroxybutyrate, and acetone are ketone bodies that can diffuse from the liver into general circulation and be used as fuels by peripheral tissues [49]. Some tissues such as brain, muscle, and kidney use acetoacetate in preference to glucose to produce energy [49]–[51]. Because torpid bats have low blood glucose [52], [53] but high acetoacetate levels in long winter fast (Table 2), it is conceivable that acetoacetate and fumarate are used as fuels during hibernation [54]. This idea agrees with the general concept that hibernating mammals utilize ketone bodies as their energy supplies [55]. An additional advantage for the utilization of acetoacetate during hibernation is protection of neuronal cells form oxidative glutamate toxicity [56].

A very striking finding of this study is that the expressions of PAH, HGD, and FAH were simultaneously upregulated during hibernation in two different species of bats, *Myotis ricketti* (Yangochiroptera) and *Rhinolophus ferrumequinum* (Yinpterochiroptera) (Figure 3B, species trees). Although both of these bats are hibernators, they are distantly related. This finding suggests that this co-upregulation is highly correlated with hibernation regardless of species (Figures 3 and S4). Further support for this notion is the result of a recent study showing that the expressions of *HPD* and *FAH* mRNA are much higher in the heart of golden-mantled ground squirrels (*Spermophilus lateralis*) during late torpor [57]. Furthermore, increased levels of plasma phenylalanine and tyrosine are found in hibernating ground squirrels upon arousal [58]. It has also been found that the levels of aromatic amino acids (tyrosine, phenylalanine, and tryptophan) vary significantly in the liver of 13-lined ground squirrels (*Spermophilus tridecemlineatus*) in different stages of hibernation [14]. Based on these observations and our findings of decreased phenylalanine concentration in torpid bats (Table 2), we hypothesized that phenylalanine and tyrosine catabolism is active in small hibernators during hibernation and thus the selective pressure of this pathway in hibernators and non-hibernators would be different due to the differences in their metabolic approaches.

To test this evolutionary assumption, we calculated the ω values of *PAH*, *HPD*, *HGD* and *FAH* genes in eight to eleven species of bats to determine the selective pressure acting on each gene. Consistent with our hypothesis, significant differences between hibernators and non-hibernators in selective pressure on *PAH*, *HPD*, and *HGD* were identified with a stronger selective constraint (i.e., lower d_N_/d_S_ value) in hibernating bats (Figure 4). One possible explanation for this difference is that hibernating bats (e.g., *M. ricketti* and *R. ferrumequinum*) live in relatively high latitudes where diurnal and seasonal temperature variations are more extreme. In contrast, non-hibernating bats (e.g., *Cynopterus sphinx*, *Pteropus vampyrus*, and *Rousettus leschenaultii)* live in lower latitudes with milder weather and thus less selective pressure. A stronger selective constraint driven by environmental stresses (e.g., low temperature or food shortage) may affect expression patterns, functions, and structures of these enzymes. As shown in Figure 4, all the enzymes examined had a strong purifying selection (ω<0.3). Because a stronger purifying selection reflects a higher functional constrain on the protein, these results suggest the functional importance of these enzymes in hibernation.

Further support for the functional importance of PAH, HPD, HGD, and FAH in hibernating bats is the observation that hibernators had more conserved amino acid positions than non-hibernators in each of these enzymes (Figures 5, S5, and S6). Among the amino acid positions with sequence variations, 50 positions in PAH, 36 positions in HPD, 12 positions in HGD, and 19 positions in FAH are 100% conserved in all hibernators. The amino acid residues at these positions of the four enzymes in non-hibernators, however, are either different from those of hibernators, or vary among the non-hibernators (Figures 5, S5, and S6). Of great importance is that most amino acid residues at these conserved positions play important roles in the regulation of enzyme activity or maintenance of enzyme structure (Figures 5, S5, and S6). Taken together, our data reveal the essential role of phenylalanine and tyrosine metabolism in bats in adaptation to hibernation.

In addition to phenylalanine and tyrosine metabolism, we found a significant decrease in the levels of some structural proteins including MYL6, ACTG5, MRLC2, and TPM4 in torpid bats (Table 1), supporting the concept of protein consumption (14–65%) as an energy source in small hibernators during hibernation [7]–[9]. A consequence of protein consumption is elevated ammonia levels due to amino acid oxidation. To prevent ammonia accumulation to a toxic level, a down-regulation of GLUD2 or an up-regulation of carbamoyl-phosphate synthase (CPS1) that converts ammonia to urea is needed and was seen in this study (Table 1 and Figure S2). Other enzymes, such as argininosuccinate lyase (ASL) and arginase-1 (ARG1) that also participate in urea synthesis were found to remain expressed during torpor (Table 1), suggesting the occurrence of urea production. The reports of urea excretion of small hibernators during periodic arousals further support this possibility [7], [59]. Proteolytic responses are also suggested by results of IPA showing a high correlation (*P* value of overlap <10^−6^) between MYC family proteins and proliferator-activated receptor (PPARA) and hibernation (Figure 2). These transcription factors are involved in the maintenance of amino acid homeostasis [60], [61].

Another important finding in this study is the observation that the expression levels of some enzymes involved in glucose production were decreased or not changed. The blood glucose levels of torpid bats were lower than 3 mmol/L, but were increased up to 9 mmol/L within 2 hours after arousal. As shown in Table 1, the expression level of malate dehydrogenase (MDH1), that generates oxaloacetate for gluconeogenesis, was not changed in torpid bats. The expression of one isoform of propionyl-CoA carboxylase (PCCA) involved in glucose production was decreased as seen in 13-lined ground squirrels during hibernation [12]. Moreover, the expression of many glycogen phosphorylase (PYGL) isoforms that convert glycogen to glucose was decreased. In contrast, the expression level of the medium-chain acyl-CoA dehydrogenase (ACADM) that oxidize fatty acids were elevated (Table 1). These data are consistent with the general trend of energetic shifting from glycolysis to lipolysis during hibernation in other hibernators [11]–[14], [48], [62], [63].

The mechanisms that control the shift among proteolysis, lipolysis, and glycolysis pathways during hibernation are largely unknown. The reprogramming of gene expression has been hypothesized to be a mechanism [64]. It is also possible that different isoforms of enzymes carry out different functions under different physiological conditions [63]. In this study, we detected the presence of three isoforms of hydroxymethylglutaryl-CoA synthase (HMGCS2) that is a rate-limiting enzyme in ketone body generation. However, none of these isoforms showed increased levels of expression in torpid bats (Table 1). Since excessive concentration of ketone bodies would result in ketoacidosis, the lack of increase in the production of these HMGCS2 isoforms may be a mechanism by which proper levels of ketone bodies are maintained. It is conceivable that there are crosstalks between proteolytic and lipolysis pathways to ensure a sufficient energy supply during different phases of hibernation. This possibility is supported by the transcription factors such as PPARs and HNFs predicted by IPA (Figure 2) that play a role in the regulation of both proteolysis and lipolysis [60], [65]. As white adipose tissue can store triglycerides and release fatty acids to produce ketone bodies, its lipolysis is crucial for energy supply during hibernation [66], [67]. The crosstalks between the lipolysis pathway and other metabolic pathways warrant further investigations in order to fully understand the roles of various pathways in energy supply during hibernation.

## Conclusion

In the current study, we found evidence of adaptation of enzymes responsible for the catabolism of phenylalanine and tyrosine to hibernation at both DNA and protein levels. The evolutionary evidence and protein expression profiles, along with the function and structure information identified, indicate the essential roles of the pathway during hibernation of bats. Evidences that indicate detoxification of nitrogen metabolites and a possible crosstalk between proteolysis and lipolysis pathways were also observed. Further multidisciplinary studies of more metabolic pathways in more mammalian species are needed to understand the mechanisms by which the proteolytic and antiproteolytic balance is maintained and to create a complete and non-biased metabolic map to further understand mammalian hibernation.

## Supporting Information

Figure S1 **2D-gel electrophoresis of liver proteins of** ***Myotis ricketti*** **.** The second pair (**A**) and third pair (**B**) of bats in hibernation (left panel) and aroused states (right panel) are shown. Numbers on the left of the gel indicate the positions and approximate molecular mass (in kDa) of the marker proteins run on the same gel. The numbers below the gel denote the approximate pH gradient across the gel. Protein spots indicated are PAH (32), HPD (41), HGD (42–44), and FAH (46, 47).
(TIF)

Figure S2 **Expressions of GLUD2 and CPS1.** GLUD2 and CPS1 protein levels in bats, pigs, rats, and mice were determined by Western blotting. H and A represent bats in hibernation and active states, respectively. Arrows indicate the predicted molecular weight (kDa) of the proteins; the numbers in parentheses denote observed molecular weights. (**B**) Relative protein levels (y-axis) of bats are represented as mean ± SD. The lowest level of a detectable protein is considered as 10. Statistical significance was determined by one-way ANOVA. *P<0.05. **P<0.001.
(TIF)

Figure S3 **Ingenuity pathway analyses of functional pathway.** Two main interacting networks are shown. Interacting proteins are colored in white. Upregulated and down-regulated expressions are indicated in red and green, respectively.
(TIF)

Figure S4 **Expression patterns of enzymes in Phe and Tyr catabolic pathway.** PAH, HPD, HGD, and FAH protein levels in pigs, rats, mice, and female (**A,** ♀) bats were determined by Western blotting. H and A represent bats in hibernation and active states, respectively. Arrows indicate the predicted molecular weight (kDa) of the proteins; the numbers in parentheses denote observed molecular weights. Asterisk represents an unknown protein band. (**B**) Relative protein levels (y-axis) of female bats are represented as mean ± SD. The lowest level of a detectable protein is considered as 1. Species trees are drawn at the bottom of the panel to demonstrate the phylogenetic relationships among the mammalian species examined. Statistical significance was determined by one-way ANOVA (*P<0.05; **P<0.001).
(TIF)

Figure S5 **Structure based sequence alignment of PAH and HPD.** Amino acid sequences of PAH and HPD from hibernating (H) and non-hibernating (N) bats and each of their corresponding template of human (*Homo sapiens*), rat (*Rattus norvegicus*), or mouse (*Mus musculus*) are aligned. Amino acids that are conserved among hibernating bats but are different or diverged among non-hibernating ones are colored in blue and red, respectively. Orange color indicates positions conserved in non-hibernating bats but diverged among hibernating bats. (**A**) The catalytic domain of PAH consisting of residues R131-L137, L250-R253, D316-W327, and E377-I380 is denoted with an orange line. The autoregulatory region (residues 20–34) at the N-terminus is indicated by the red line, and residues S2-Q21 that stabilizes the secondary structure of the phosphorylated form of PAH are indicated by the green line. The region marked with a blue line (residues R112-T118) is the hinge region essential for the phenylalanine-modulated proteolytic cleavage, and that marked with a pink line (residues L431-K453) is the region required for the formation of a fully active enzyme tetramer. The residues indicated in triangles are the ones known to have point mutations in humans with phenylketonuria (PKU). (**B**) Amino acids H183, H266, and E349 of HPD involved in catalytic activity are marked in green. All mutations that have been reported to cause type 3 tyrosinemia in human are indicated with purple triangles.
(PDF)

Figure S6 **Structure based sequence alignment of HGD and FAH.** Amino acid sequences of HGD and FAH from hibernating (H) and non-hibernating (N) bats and each of their corresponding template of human (*Homo sapiens*), rat (*Rattus norvegicus*), or mouse (*Mus musculus*) are aligned. Amino acids that are conserved among hibernating bats but are different or diverged among non-hibernating ones are colored in blue and red, respectively. Orange color indicates positions conserved in non-hibernating bats but diverged among hibernating bats. (**A**) The active sites of HGD composed of residues F282-T299, P320-K327, and M368-K385 are denoted with the orange line. Residue H269 is critical for the trimerization of HGD and activation of the enzyme. Positions that have been reported to have missense or nonsense mutations leading to human alkaptonuria (AKU) are indicated in triangles. (**B**) Amino acids involved in the catalytic activity of FAH are indicated in green, in which D126, E199, E201, and D233 are responsible for binding of a metal ion, and T350 is for binding of a water molecule. The oxyanion hole formed by the side chains of R237, Q240, and K253 stabilizes the transition state during enzymatic reaction. The H133/E364 catalytic dyad is drawn in pink. Residues M149-Y190 denoted by the gray line are located in the hydrophobic region of C-terminus which is buried in the inter surface of the FAH dimer. The amino acids indicated in triangles are the ones known to cause type 1 tyrosinemia in humans if they are mutated.
(PDF)

Table S1 **Accession numbers of nucleotide sequences submitted and those obtained from Ensemble.**
(TIF)

Table S2 **Primers used for amplification of** ***PAH*** **,** ***HPD*** **,** ***HGD*** **, and** ***FAH*** **genes.**
(TIF)

Table S3 **ID numbers of known structures used for molecular simulation.**
(TIF)
