# Supplementary figures and images for: Adaptation of Phenylalanine and Tyrosine Catabolic Pathway to Hibernation in Bats
Source: PLoS One. 2013 Apr 19;8(4):e62039. doi: 10.1371/journal.pone.0062039 (PMC3631164; doi:10.1371/journal.pone.0062039)

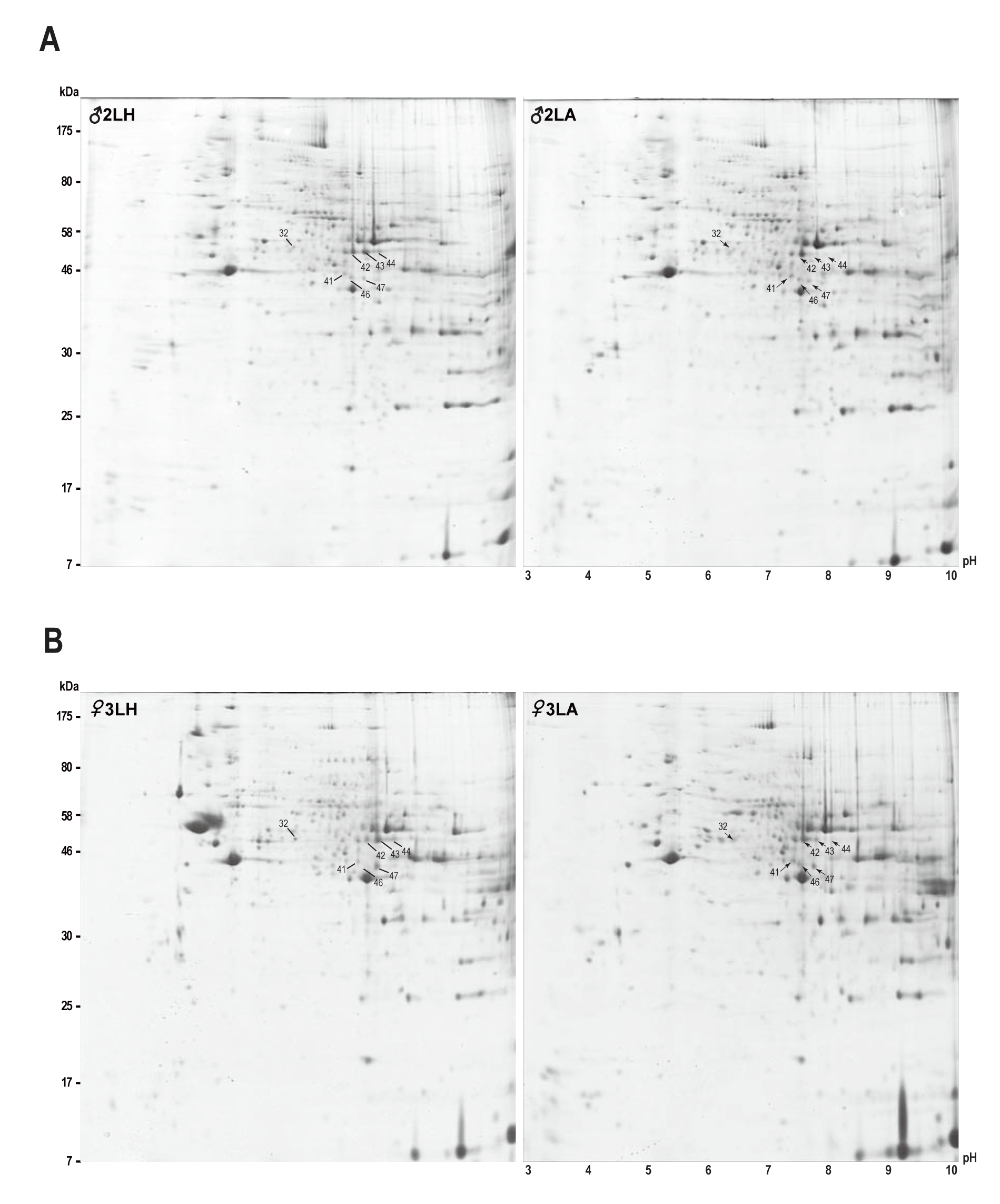

Supplement: Figure S1 — 2D-gel electrophoresis of liver proteins of Myotis ricketti . The second pair (A) and third pair (B) of bats in hibernation (left panel) and aroused states (right panel) are shown. Numbers on the left of the gel indicate the positions and approximate molecular mass (in kDa) of the marker proteins run on the same gel. The numbers below the gel denote the approximate pH gradient across the gel. Protein spots indicated are PAH (32), HPD (41), HGD (42–44), and FAH (46, 47). (TIF) [file pone.0062039.s001.tif]

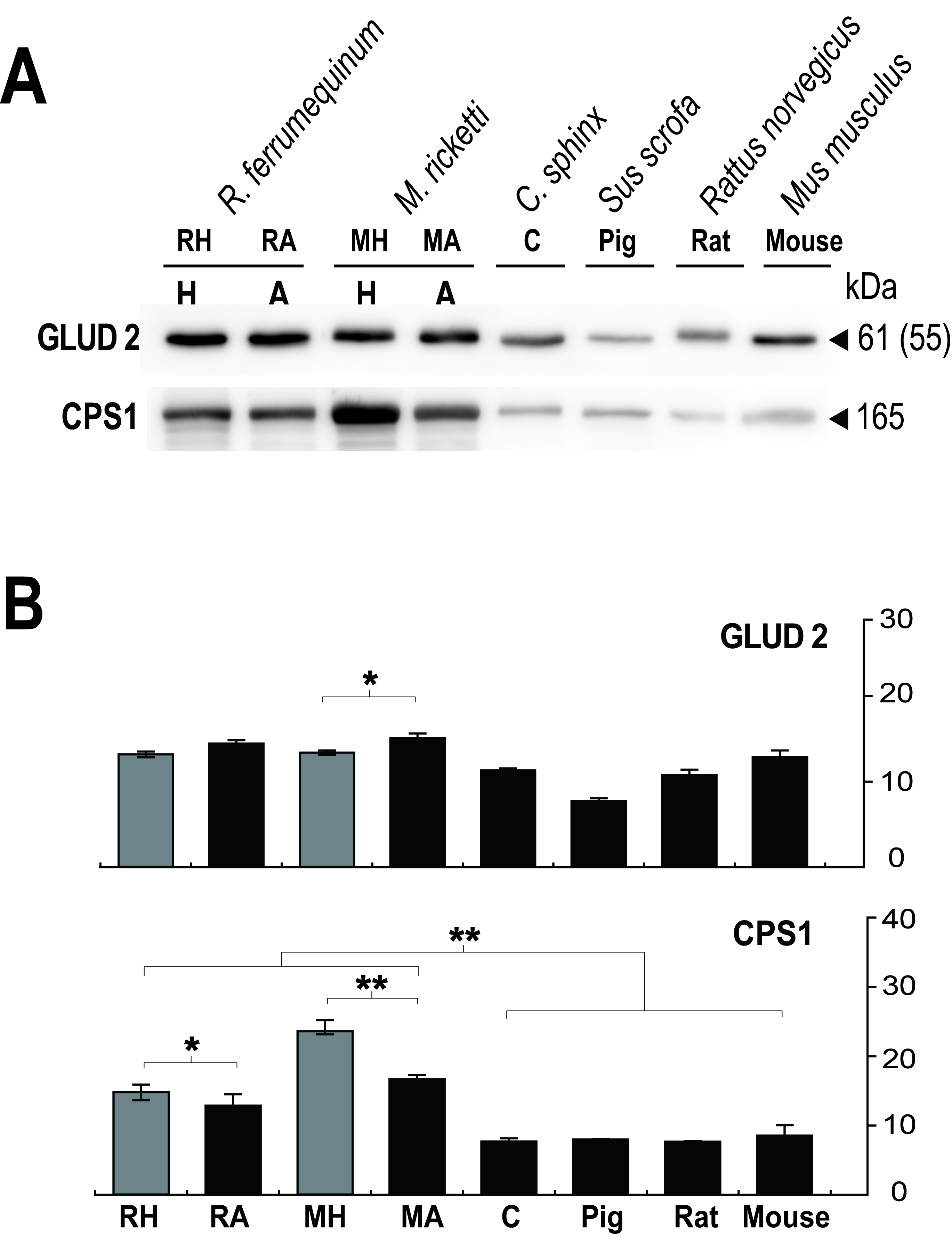

Supplement: Figure S2 — Expressions of GLUD2 and CPS1. GLUD2 and CPS1 protein levels in bats, pigs, rats, and mice were determined by Western blotting. H and A represent bats in hibernation and active states, respectively. Arrows indicate the predicted molecular weight (kDa) of the proteins; the numbers in parentheses denote observed molecular weights. (B) Relative protein levels (y-axis) of bats are represented as mean ± SD. The lowest level of a detectable protein is considered as 10. Statistical significance was determined by one-way ANOVA. *P<0.05. **P<0.001. (TIF) [file pone.0062039.s002.tif]

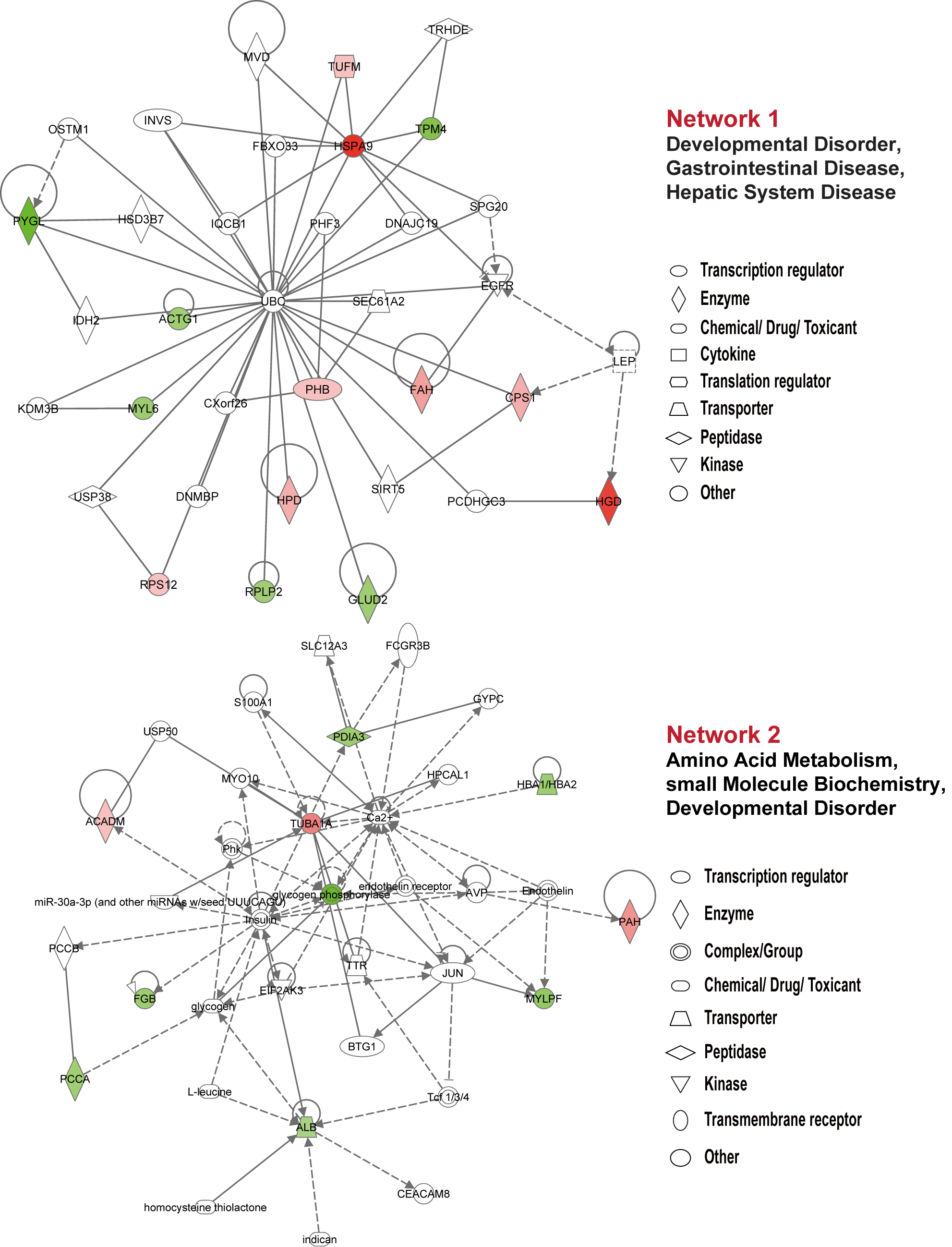

Supplement: Figure S3 — Ingenuity pathway analyses of functional pathway. Two main interacting networks are shown. Interacting proteins are colored in white. Upregulated and down-regulated expressions are indicated in red and green, respectively. (TIF) [file pone.0062039.s003.tif]

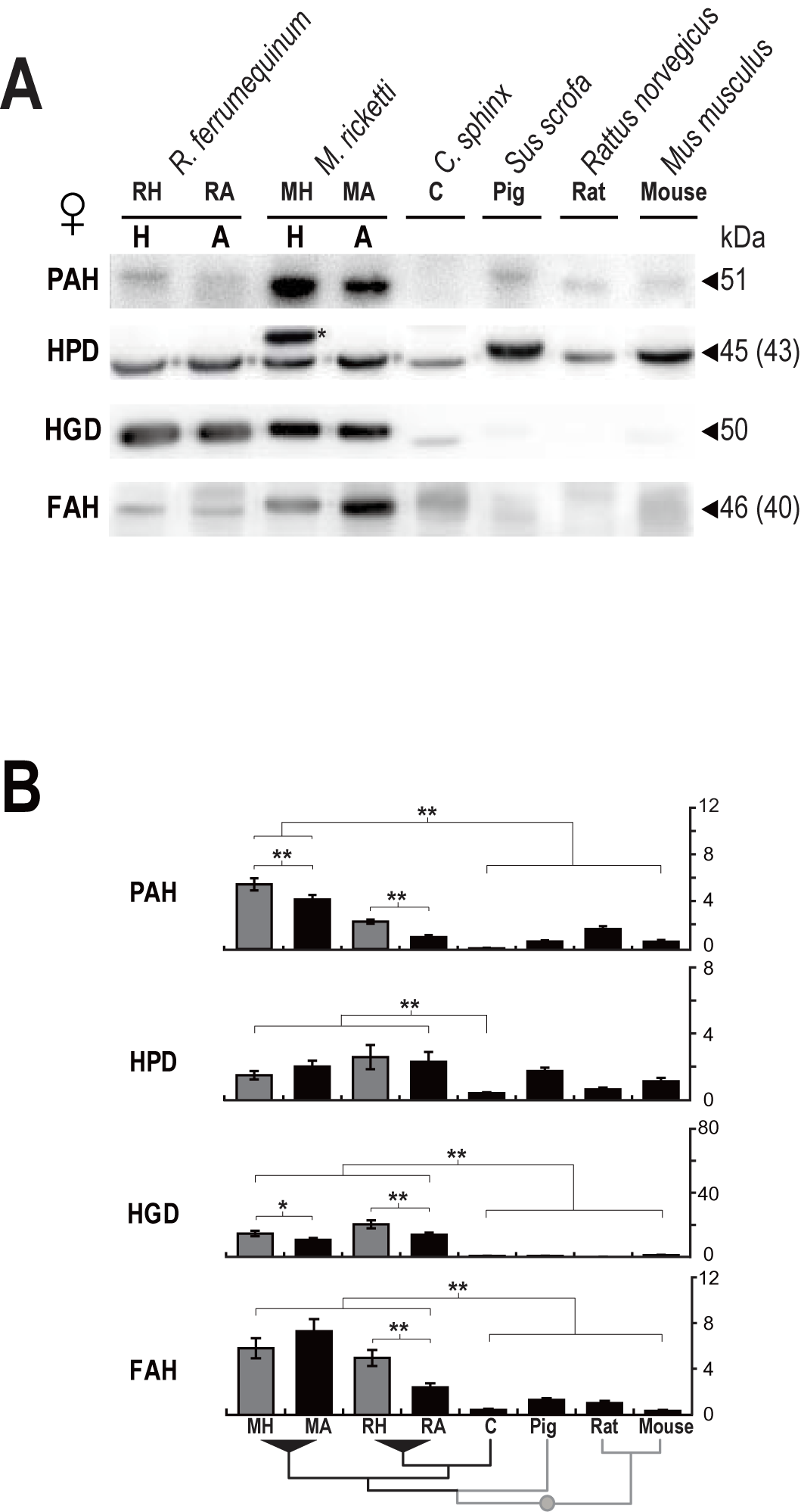

Supplement: Figure S4 — Expression patterns of enzymes in Phe and Tyr catabolic pathway. PAH, HPD, HGD, and FAH protein levels in pigs, rats, mice, and female (A, ♀) bats were determined by Western blotting. H and A represent bats in hibernation and active states, respectively. Arrows indicate the predicted molecular weight (kDa) of the proteins; the numbers in parentheses denote observed molecular weights. Asterisk represents an unknown protein band. (B) Relative protein levels (y-axis) of female bats are represented as mean ± SD. The lowest level of a detectable protein is considered as 1. Species trees are drawn at the bottom of the panel to demonstrate the phylogenetic relationships among the mammalian species examined. Statistical significance was determined by one-way ANOVA (*P<0.05; **P<0.001). (TIF) [file pone.0062039.s004.tif]

## A. PAH

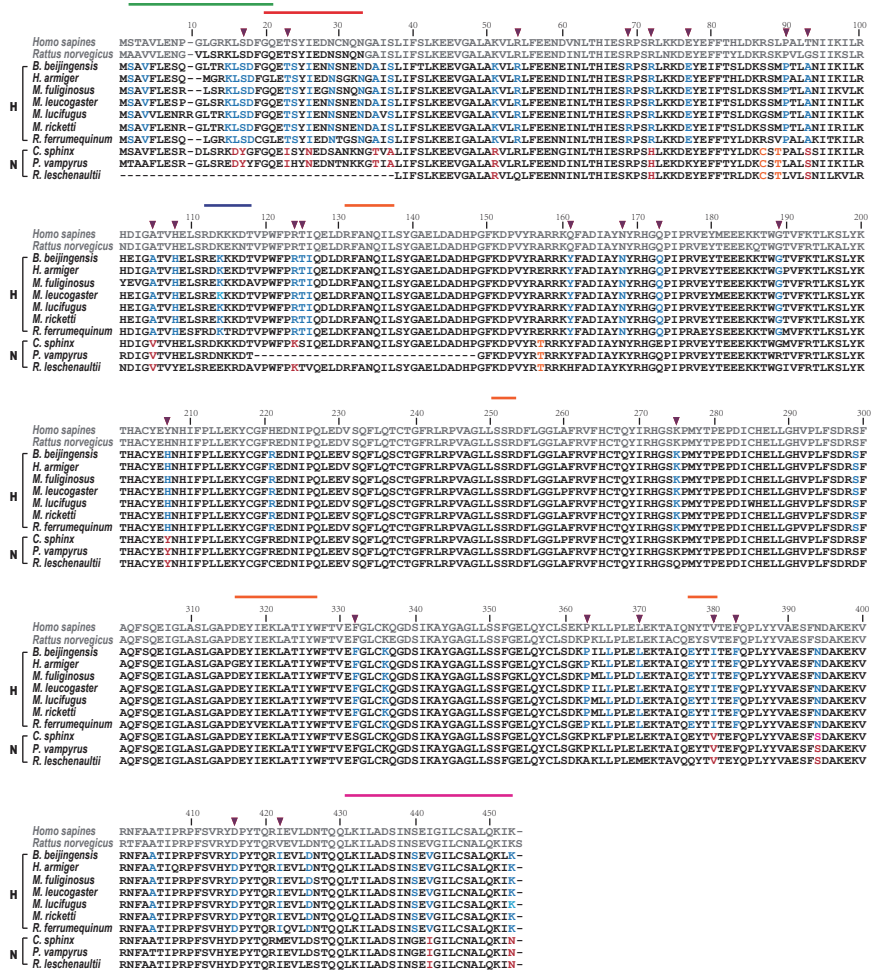

## B. HPD

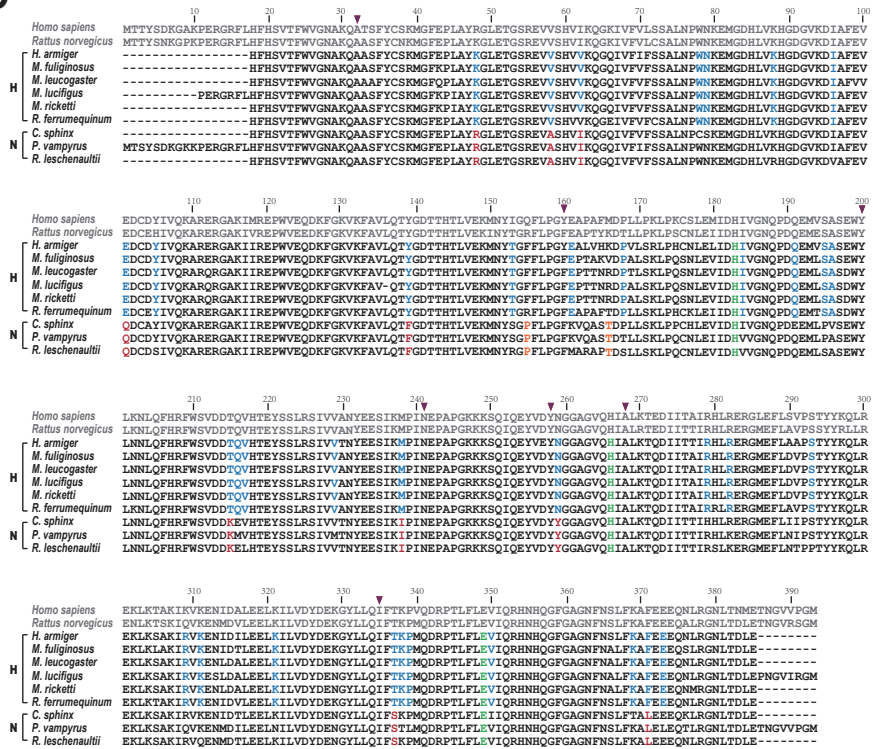

Supplement: Figure S5 — Structure based sequence alignment of PAH and HPD. Amino acid sequences of PAH and HPD from hibernating (H) and non-hibernating (N) bats and each of their corresponding template of human (Homo sapiens), rat (Rattus norvegicus), or mouse (Mus musculus) are aligned. Amino acids that are conserved among hibernating bats but are different or diverged among non-hibernating ones are colored in blue and red, respectively. Orange color indicates positions conserved in non-hibernating bats but diverged among hibernating bats. (A) The catalytic domain of PAH consisting of residues R131-L137, L250-R253, D316-W327, and E377-I380 is denoted with an orange line. The autoregulatory region (residues 20–34) at the N-terminus is indicated by the red line, and residues S2-Q21 that stabilizes the secondary structure of the phosphorylated form of PAH are indicated by the green line. The region marked with a blue line (residues R112-T118) is the hinge region essential for the phenylalanine-modulated proteolytic cleavage, and that marked with a pink line (residues L431-K453) is the region required for the formation of a fully active enzyme tetramer. The residues indicated in triangles are the ones known to have point mutations in humans with phenylketonuria (PKU). (B) Amino acids H183, H266, and E349 of HPD involved in catalytic activity are marked in green. All mutations that have been reported to cause type 3 tyrosinemia in human are indicated with purple triangles. (PDF) [file pone.0062039.s005.pdf]

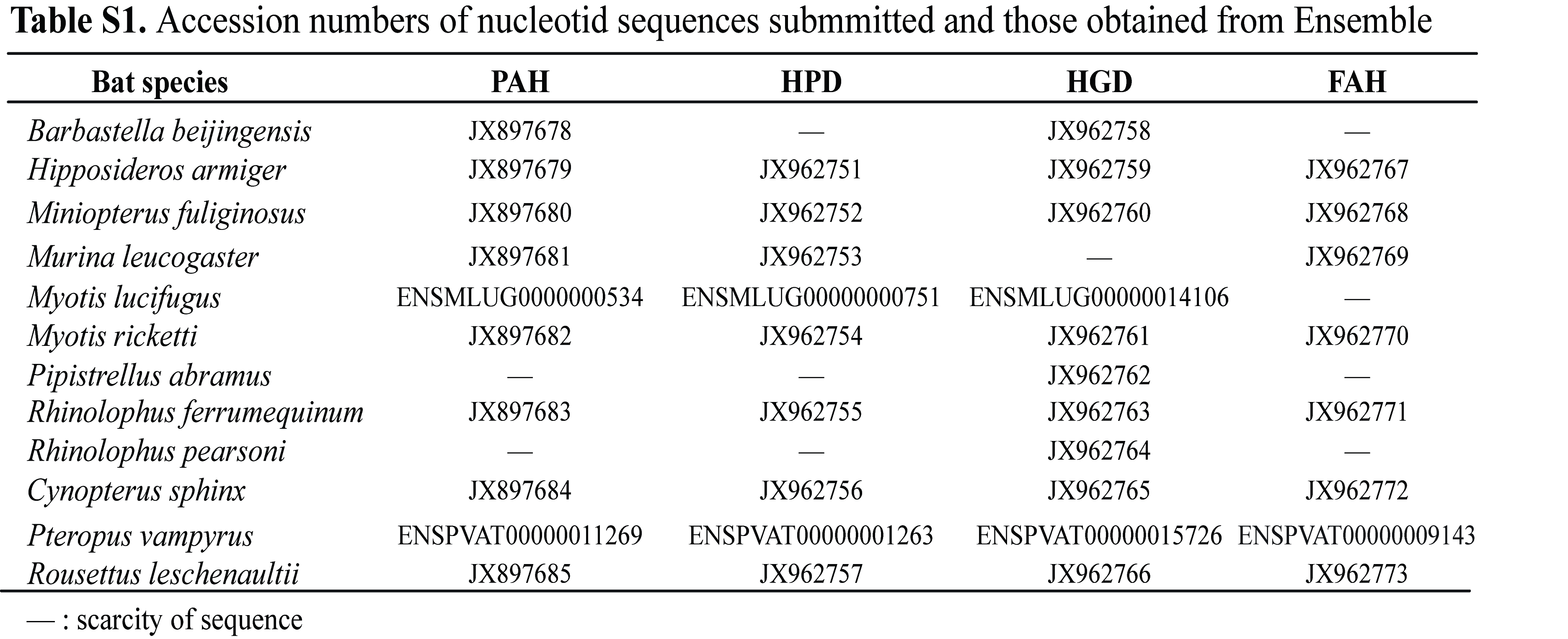

Supplement: Table S1 — Accession numbers of nucleotide sequences submitted and those obtained from Ensemble. (TIF) [file pone.0062039.s007.tif]

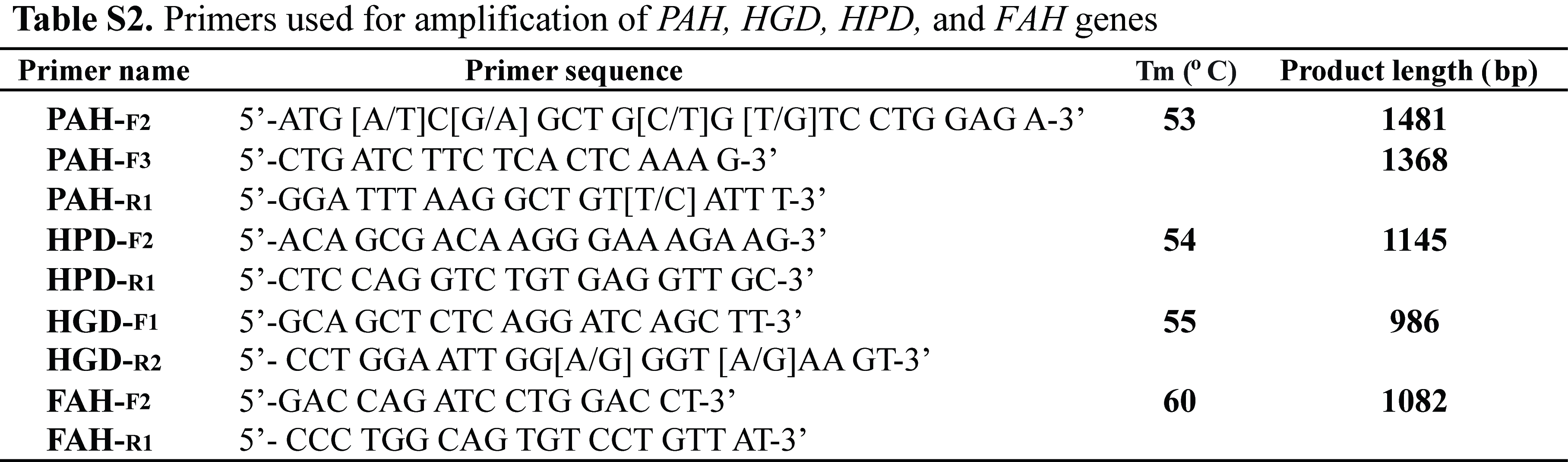

Supplement: Table S2 — Primers used for amplification of PAH , HPD , HGD , and FAH genes. (TIF) [file pone.0062039.s008.tif]

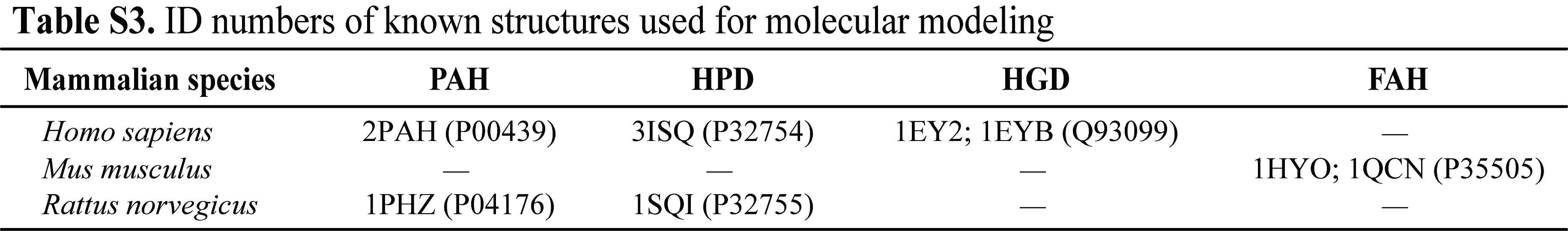

Supplement: Table S3 — ID numbers of known structures used for molecular simulation. (TIF) [file pone.0062039.s009.tif]
