# Supplementary material for: Adaptation of Phenylalanine and Tyrosine Catabolic Pathway to Hibernation in Bats
Source: PLoS One. 2013 Apr 19;8(4):e62039. doi: 10.1371/journal.pone.0062039 (PMC3631164; doi:10.1371/journal.pone.0062039)
Supplement: Figure S6 — Structure based sequence alignment of HGD and FAH. Amino acid sequences of HGD and FAH from hibernating (H) and non-hibernating (N) bats and each of their corresponding template of human (Homo sapiens), rat (Rattus norvegicus), or mouse (Mus musculus) are aligned. Amino acids that are conserved among hibernating bats but are different or diverged among non-hibernating ones are colored in blue and red, respectively. Orange color indicates positions conserved in non-hibernating bats but diverged among hibernating bats. (A) The active sites of HGD composed of residues F282-T299, P320-K327, and M368-K385 are denoted with the orange line. Residue H269 is critical for the trimerization of HGD and activation of the enzyme. Positions that have been reported to have missense or nonsense mutations leading to human alkaptonuria (AKU) are indicated in triangles. (B) Amino acids involved in the catalytic activity of FAH are indicated in green, in which D126, E199, E201, and D233 are responsible for binding of a metal ion, and T350 is for binding of a water molecule. The oxyanion hole formed by the side chains of R237, Q240, and K253 stabilizes the transition state during enzymatic reaction. The H133/E364 catalytic dyad is drawn in pink. Residues M149-Y190 denoted by the gray line are located in the hydrophobic region of C-terminus which is buried in the inter surface of the FAH dimer. The amino acids indicated in triangles are the ones known to cause type 1 tyrosinemia in humans if they are mutated. (PDF) [file pone.0062039.s006.pdf]

A. HGD

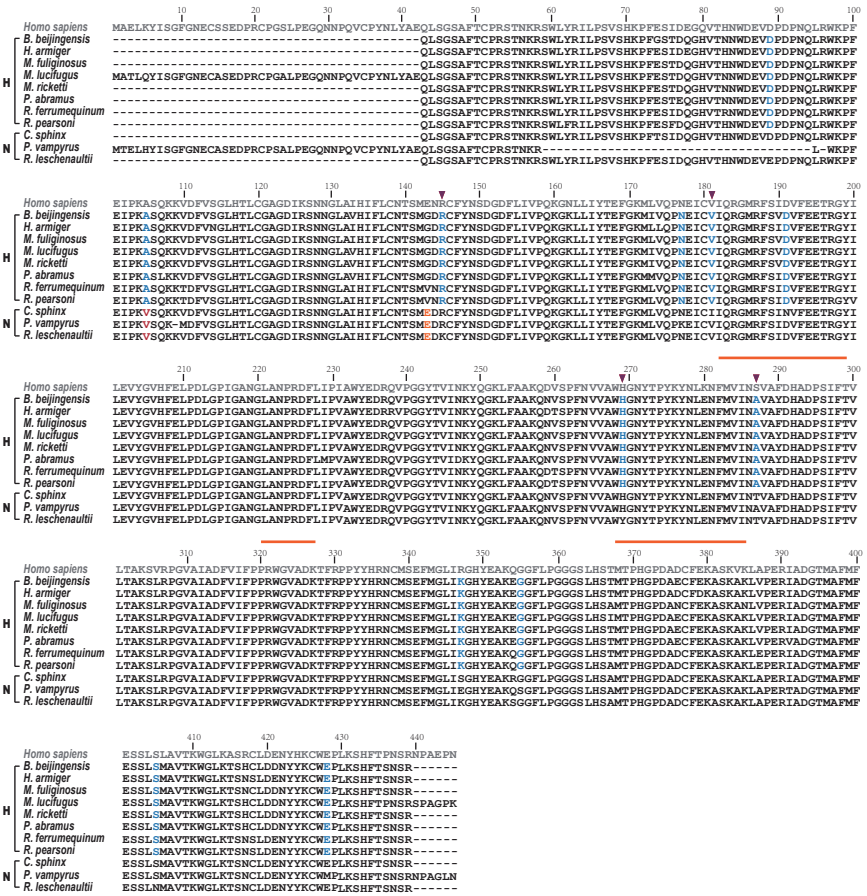
Homo sapiens
B. beijingensis
H. armiger
M. fuliginosus
M. lucifugus
M. ricketti
P. abramus
R. ferrumequinum
R. pearsoni
C. sphinx
P. vampyrus
R. leschenaultii
H
N

B. FAH

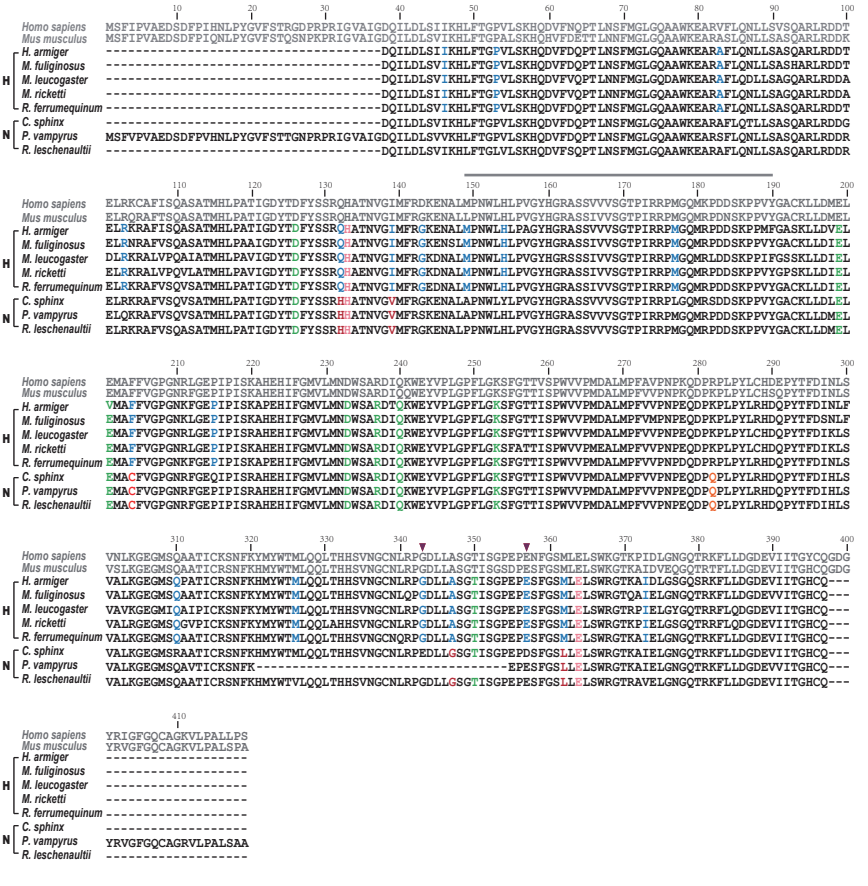
Homo sapiens
Mus musculus
H. armiger
M. fuliginosus
M. leucogaster
M. ricketti
R. ferrumequinum
C. sphinx
P. vampyrus
R. leschenaultii
H
N
